# Human Leg Model Predicts Muscle Forces, States, and Energetics during Walking

**DOI:** 10.1371/journal.pcbi.1004912

**Published:** 2016-05-13

**Authors:** Jared Markowitz, Hugh Herr

**Affiliations:** MIT Media Lab, Massachusetts Institute of Technology, Cambridge, Massachusetts, United States of America; Johns Hopkins University, UNITED STATES

## Abstract

Humans employ a high degree of redundancy in joint actuation, with different combinations of muscle and tendon action providing the same net joint torque. Both the resolution of these redundancies and the energetics of such systems depend on the dynamic properties of muscles and tendons, particularly their force-length relations. Current walking models that use stock parameters when simulating muscle-tendon dynamics tend to significantly overestimate metabolic consumption, perhaps because they do not adequately consider the role of elasticity. As an alternative, we posit that the muscle-tendon morphology of the human leg has evolved to maximize the metabolic efficiency of walking at self-selected speed. We use a data-driven approach to evaluate this hypothesis, utilizing kinematic, kinetic, electromyographic (EMG), and metabolic data taken from five participants walking at self-selected speed. The kinematic and kinetic data are used to estimate muscle-tendon lengths, muscle moment arms, and joint moments while the EMG data are used to estimate muscle activations. For each subject we perform an optimization using prescribed skeletal kinematics, varying the parameters that govern the force-length curve of each tendon as well as the strength and optimal fiber length of each muscle while seeking to simultaneously minimize metabolic cost and maximize agreement with the estimated joint moments. We find that the metabolic cost of transport (MCOT) values of our participants may be correctly matched (on average 0.36±0.02 predicted, 0.35±0.02 measured) with acceptable joint torque fidelity through application of a single constraint to the muscle metabolic budget. The associated optimal muscle-tendon parameter sets allow us to estimate the forces and states of individual muscles, resolving redundancies in joint actuation and lending insight into the potential roles and control objectives of the muscles of the leg throughout the gait cycle.

## Author Summary

Neuromuscular systems often employ redundancy in joint actuation, with different combinations of muscle and tendon action producing the same net joint torque. Both the resolution of these redundancies and the energetics of such systems depend strongly on the force-length relations of muscles and tendons. Many human walking models fail to properly account for elasticity by failing to scale muscle-tendon parameters for different individuals, relying instead on stock values taken from cadaver studies. This can result in inaccurate estimates of metabolic consumption as well as of the forces and states of individual muscles. Instead we estimate muscle-tendon parameters using a data-driven optimization procedure, testing the hypothesis that the human leg has evolved to maximize the metabolic efficiency of walking at self-selected speed. We find that the experimentally observed metabolic consumption can be matched with reasonable joint torque fidelity through the addition of a single constraint on the per-muscle metabolic budget. The associated muscle-tendon parameter sets were used to compute muscle force and state estimates, lending insight into potential roles and control objectives of the major muscles of the leg throughout the gait cycle.

## Introduction

Human walking relies on a complex interplay of several physiological systems, with each exhibiting some degree of redundancy. The nervous system directs muscle contraction while receiving input from many different neural pathways. Muscles work together to produce motion, but different combinations of muscle action can produce the same net torque at a given joint. Tendons provide the interface between muscle and bone, but the energy transferred to the skeleton can come from either the active muscle or the compliance of the tendon. Understanding how humans resolve these redundancies has been a long-standing problem in the fields of neuroscience and biomechanics [1, 2].

Knowledge of how the neuromuscular system allocates load during a given task would provide insight into the control objectives that govern its actions. Potential objectives (reviewed in [3]) include joint trajectory planning or minimization of metabolic energy consumption, active muscle volume, or muscle fatigue. However without an adequate understanding of the roles of each component of the system, such control hypotheses remain mere speculation.

The roles of individual muscles and tendons in producing motion depend on the neural drive to the muscles and on the force generation properties of both the muscles and the tendons. Several modes of experimental observations provide glimpses of these elements. Electromyography (EMG) can be used to quantify the neural drive to individual muscles, revealing which muscles contribute to a given movement and giving some measure of intensity [4–6]. However it is limited by signal variability, measurement artifacts, and an inexact mapping to physiology and muscle force. Ultrasound probes have recently been used to image the individual motions of some distal leg muscles and tendons *in vivo* [7–13], but are practical only for small muscles and limited tasks. Motion capture can be combined with a knowledge of anatomy to infer the net movement of muscle-tendon units under much more general circumstances [14]; however breaking the resulting movement profiles into individual muscle and tendon contributions requires knowledge of often unavailable force generation parameters of muscles and tendons. These parameters are typically estimated through cadaver studies, but the scaling of the relevant quantities among different muscles and subjects (not to mention the differences with living specimens) is not well understood [14–16] and can have a significant impact on the resulting modeled dynamics [15, 16].

Given the incomplete view afforded by current experimental measures, a unifying theoretical framework that combines the available data modes into a model of neuromuscular function is desirable. Two primary approaches have been taken to address this issue in walking: optimal control and optimal design.

Human walking studies based on optimal control [17, 18] model the morphology of leg muscle tendon units (MTUs) using literature-based estimates. They infer muscle activation through optimization, choosing control objectives such as metabolic energy minimization and/or motion tracking. They have been successful in predicting joint moments, joint torques, and ground reaction forces but often significantly overestimate the metabolic cost required for locomotion [17, 19]. While it is clear that the neural control of the biological system is optimized in some way, it may be infeasible to determine the true objective function for this approach. Many different muscle activation combinations can produce similar muscle torque values, and several different unknown control objectives and neurological factors may contribute at once. Further the underlying uncertainty in the muscle-tendon morphology may result in the excess metabolic cost observed, as improper leveraging of tendon compliance would affect muscle force and state and therefore metabolic estimates.

Human walking models based on optimal design utilize the efficiency gains that can be made through MTU parameter tuning. As Lichtwark and Wilson showed [20], experimentally observed muscle-tendon strains may be predicted by maximizing the efficiency of isolated MTUs. This result likely stems from the well-documented ability of tendon to enable muscle to operate economically [21, 22]. Krishnaswamy and Herr [23] further explored the potential of optimal design, estimating the torque breakdown of the muscles spanning the ankle during the stance phase of walking at self-selected speed. This work used EMG signals to estimate muscle activation during walking and developed an optimization framework based on the assumption that the morphology of the muscle-tendon units spanning the ankle has evolved to minimize the metabolic cost required for walking at self-selected speed. Its results indicated that one solution set is able to match both human metabolics and kinetics, demonstrating an efficient load-sharing amongst the plantar flexors that qualitatively matched available experimental data.

**Fig 1:**
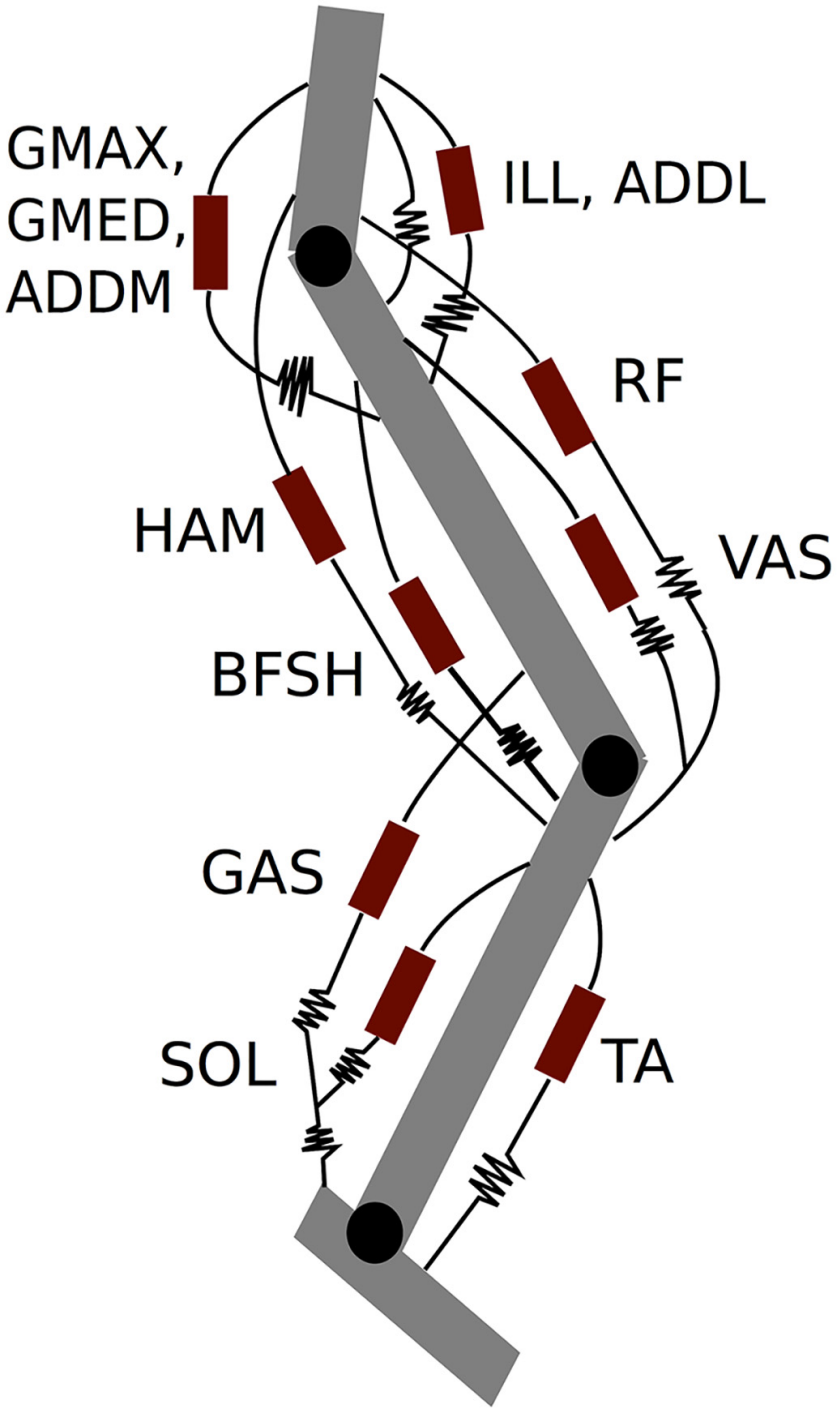
System model. The red rectangles indicate Hill type muscles while the crunched lines represent compliant elements. All compliant elements are tendons in series with muscle except for the hip flexor ligament, which provides a passive flexion moment at the hip.

**Fig 2:**
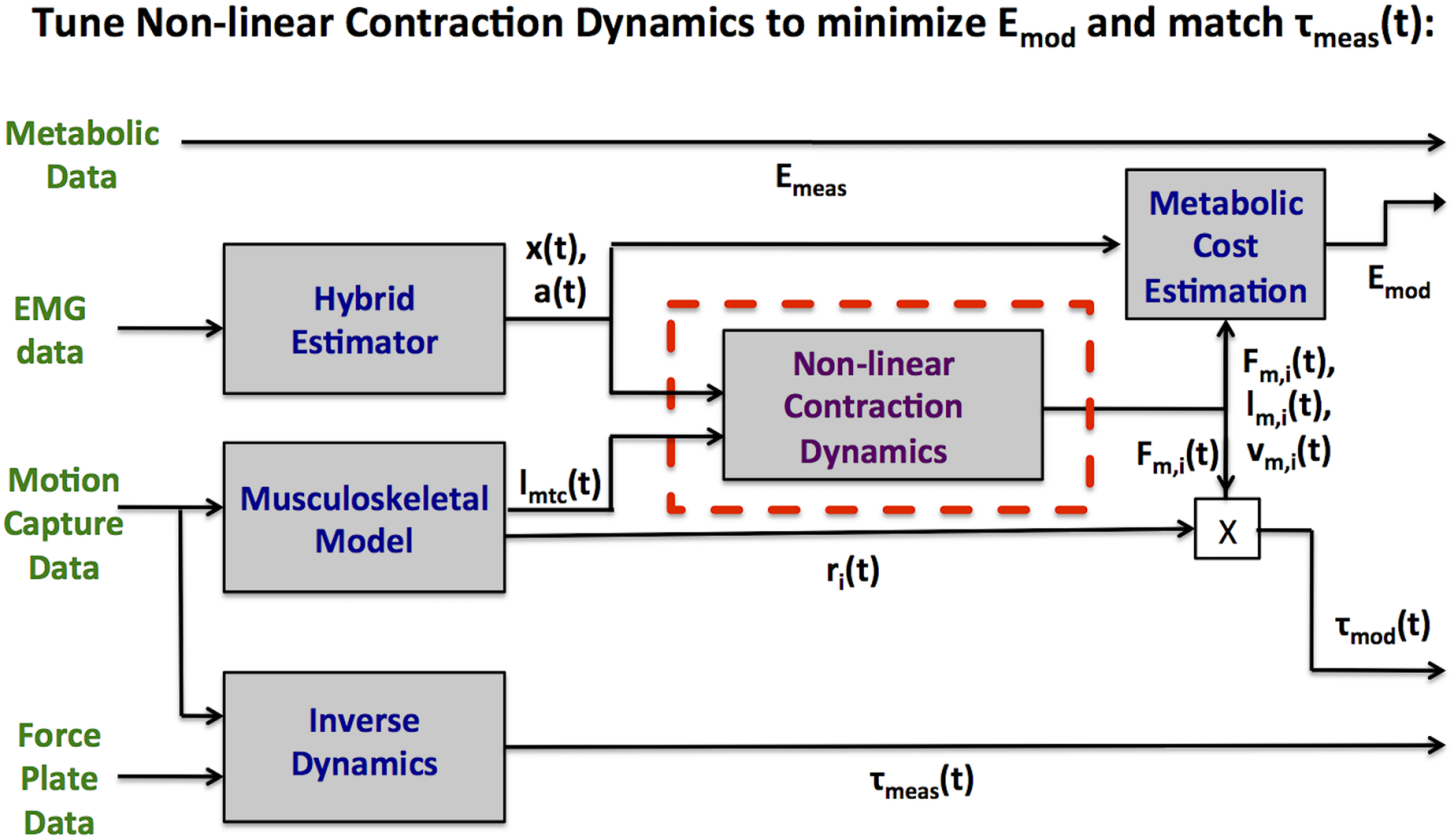
Muscle-tendon system identification procedure. The Non-linear Contraction Dynamics box comprises Hill-type representations of all muscles in the model as well as non-linear tendon dynamics.

In this work we further the study of [23], modifying and extending it to permit investigation of the full leg. We collected kinematic, kinetic, electromyographic, and metabolic data from five subjects walking at self-selected speed and used them to perform optimizations with prescribed skeletal kinematics. We varied the parameters that determine where each muscle operates on its force-length curve as well as those that shape the force-length relation of each tendon, seeking to simultaneously minimize metabolic cost and maximize agreement with the observed joint moments. Each muscle in the model was modeled as Hill-type [19, 24] and driven by activation estimates produced from EMG data. Overviews of the system model and procedure are shown in Figs 1 and 2, respectively. We found that the correct metabolic consumption can be matched with reasonable fidelity in the modeled joint torque through application of a single constraint on the per-muscle metabolic budget. The resulting optimal parameter sets were used to compute muscle force and state, with fascicle length profiles being compared to available experimental measurements.

Our results are organized as follows. First we provide our estimated muscle activation profiles, discussing their quality and implications. Second we display the results of our dual objective optimization problem and summarize the methodology used for choosing one optimal solution for each subject. Third we evaluate the optimal solutions, producing estimates of energetic variables and muscle state. We compare these results with available experimental measures, finding quantitative agreement with metabolic data and qualitative agreement with muscle fascicle length data.

## Results

### Muscle Activation Estimation

**Fig 3:**
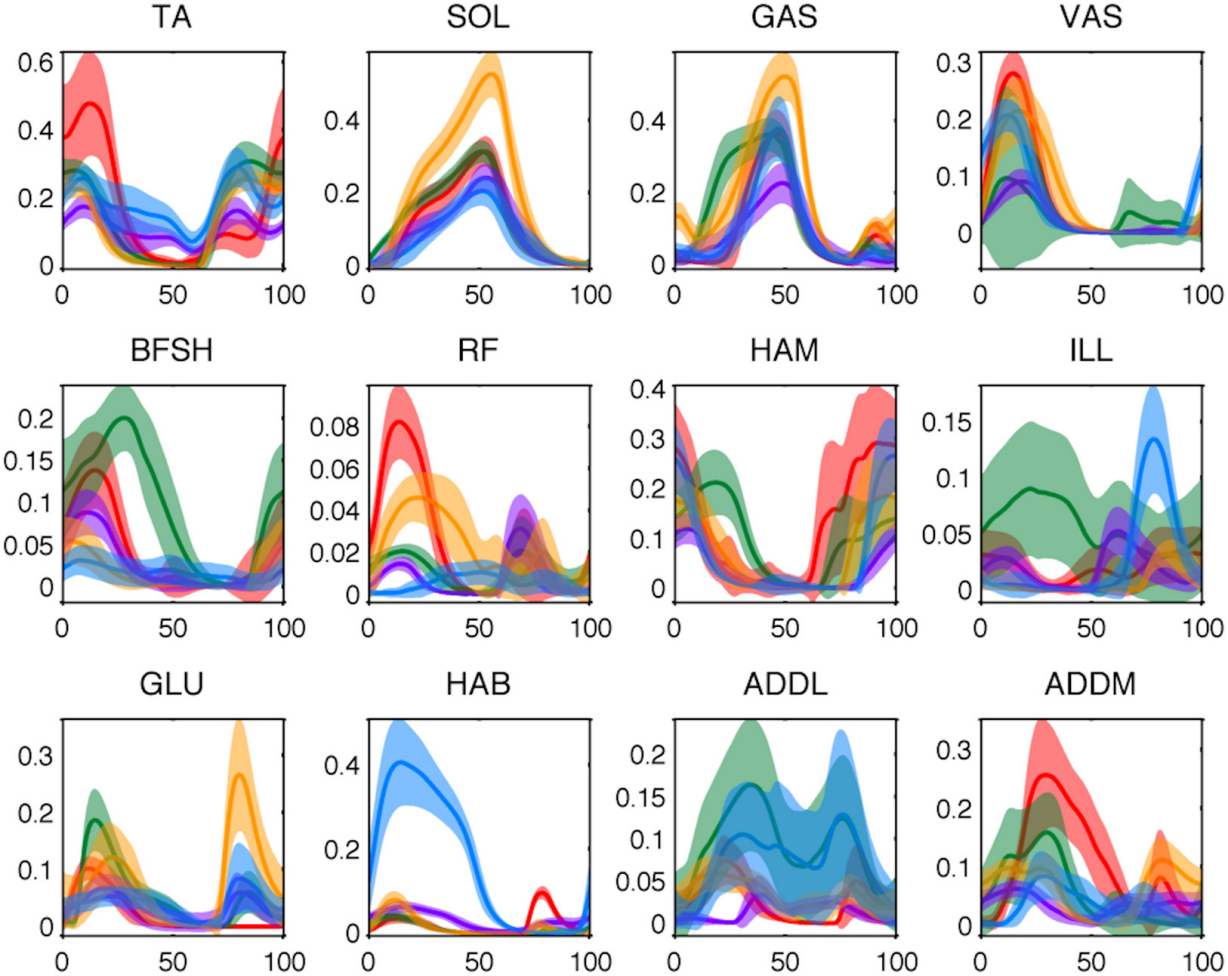
Mean activation trajectories for all muscles of all subjects at the walking speed where Metabolic Cost of Transport (MCOT) is minimal. Each color represents one participant and the shaded regions represent ± one standard deviation around the average profile.

Muscle activation provides a scaling factor for the active force generation capability of a given muscle at a given time. As described in Methods, we applied a hybrid approach similar to that of [23] to estimate muscle activation from surface EMG measurements of five participants during walking. A Bayesian algorithm first proposed by Sanger [25] was tuned (as described in Methods) and used to perform a hidden state estimation that effectively determined the neural excitation of each muscle. This method was chosen over more conventional bandpass filtering methods [4–6] because the slightly delayed timing of the profiles it produced more easily allows the production of the observed joint torques. We elaborate on this point further in the Discussion section. The estimated neural excitation was then passed to a shaping filter [26, 27] that represents muscle activation dynamics. The resulting average profiles are plotted in Fig 3. As can be seen from this plot, significant variations occurred (both within and among subjects) in the activation estimates obtained from the muscles spanning the hip. This lack of consistency was likely due to some combination of the relatively large depth of these muscles beneath the skin, motion artifacts, and the relative inaccessibility of the area. To prevent this from compromising the ensuing analysis, neural excitation profiles from the wire electrode experiments of [28] were used for the monoarticular muscles spanning the hip. These neural excitation profiles were passed to the activation dynamics from [26, 27] for temporal consistency and subsequently used as model input along with the data-based activation estimates from all other muscles. Further details about this procedure are given in Methods and S1 Text. While there is no available ground truth to compare our activation estimates to, we note that the time dependence of the results (when normalized to percent gait cycle) were relatively invariant across trial and subject. The profiles follow the expected build up and decay time scales of muscle activation and, as can be seen in the following, allow the model to produce realistic kinetic and metabolic results.

### Muscle-Tendon Parameter Identification

The estimated muscle activations *a*(*t*) as well as joint kinematics *θ*_*joint*_(*t*) derived from motion capture data were used to actuate the full leg model shown in Fig 1 according to the scheme shown in Fig 2. The leg muscle-tendon model *M* was specified by a set $\vec{m}$ of morphological parameters that describe the force generation characteristics of each MTU as well as two parameters that enable passive force generation by the iliofemoral, ischiofemoral, and pubofemoral ligaments as well as other connective tissue at the hip. These ligaments are known to prevent hip overextension and here allow for the recovery of elastic energy in the joint [18, 29]; specifically they reduce the load on the iliacus muscle around toe-off. The contributions of each MTU to $\vec{m}$ were its maximum isometric force *F*_*max*_, an overall scaling factor for tendon slack length *l*_*sl*_ and optimal muscle length *l*_*opt*_, its tendon reference strain λ_*ref*_, and its tendon shape factor *K*_*sh*_. The lumped hip flexor ligament (HFL) acted as a simple rotary spring, parameterized by spring constant *K*_*HFL*_ and engagement angle *θ*_0,*HFL*_. Each parameterization of the model generated kinetic (*τ*_*mod*_(*t*)) and metabolic (*C*) output costs:

$$\begin{array}{c} M\left(\vec{m},a\left(t\right),\theta_{joint}\left(t\right)\right)\to \left[\tau_{mod}\left(t\right),C\right]. \end{array}$$ (1)

**Fig 4:**
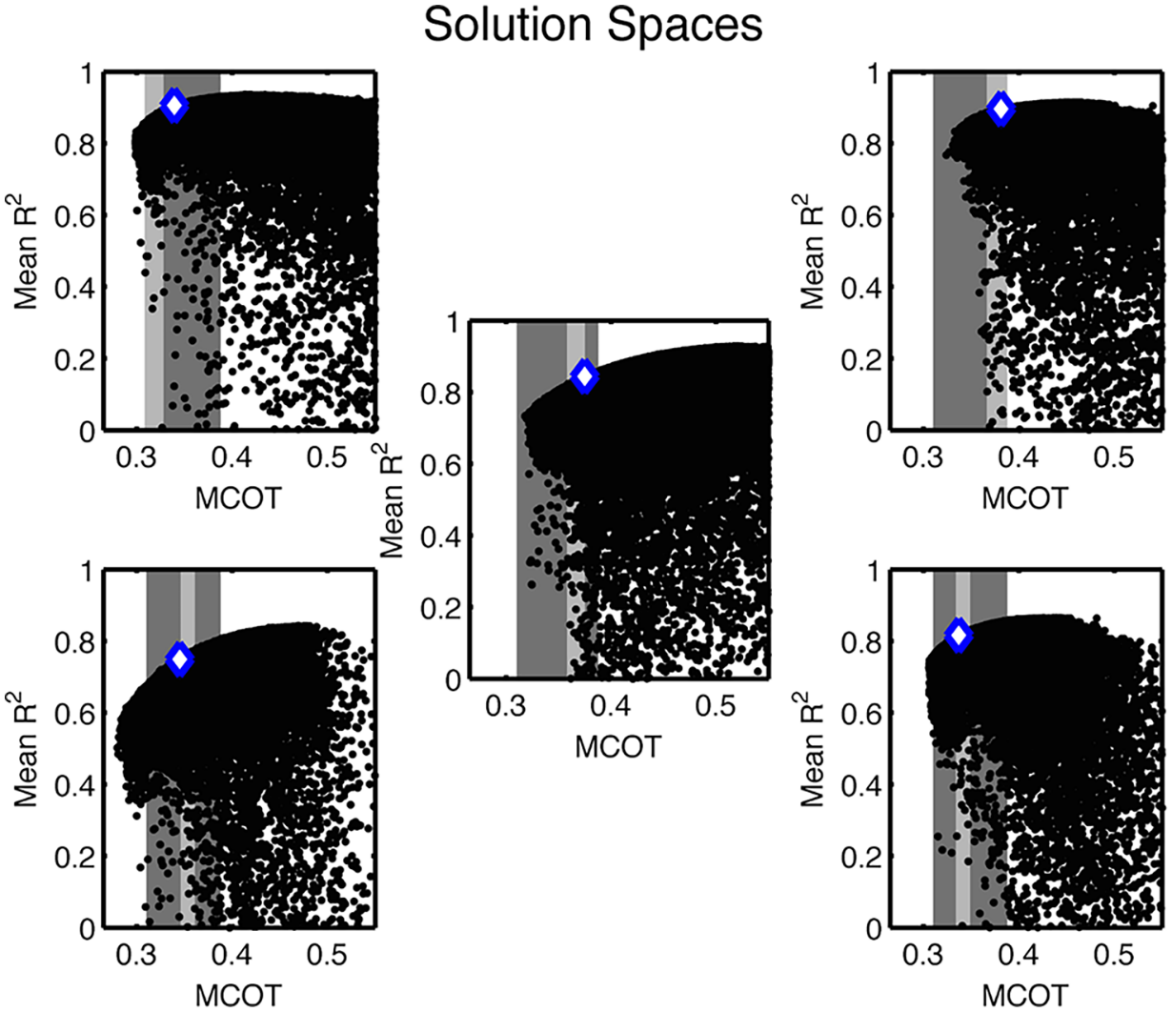
Best solution for each participant and its relation to measured metabolic cost of transport (MCOT; light gray band) and MCOT range for all participants (dark gray band). In these plots, each trial solution is represented by a dot whose x-value is its required MCOT and whose y-value is the average *R*^2^ of its kinetic predictions compared to measured ankle, knee, and hip moments.

The parameter vector $\vec{m}$ was varied using a stochastic dual objective optimization scheme [30] that simultaneously minimized metabolic cost and the difference between the computed joint moments of the data and those produced by the model. The bounds were specified as described in the Materials and Methods section; they were chosen wide enough that they were not approached by the chosen optimal parameter sets. The solution spaces for this optimization are shown in Fig 4. For each participant, the set of Pareto optimal solutions (i.e. the set of solutions where one would have to compromise on one objective to improve on the other) forms a rounded corner in the objective space. In the ideal case, this corner would be sharp and consist of one solution that optimizes both objectives. However this ideal does not typically occur in noisy (realistic) systems and does not here.

**Fig 5:**
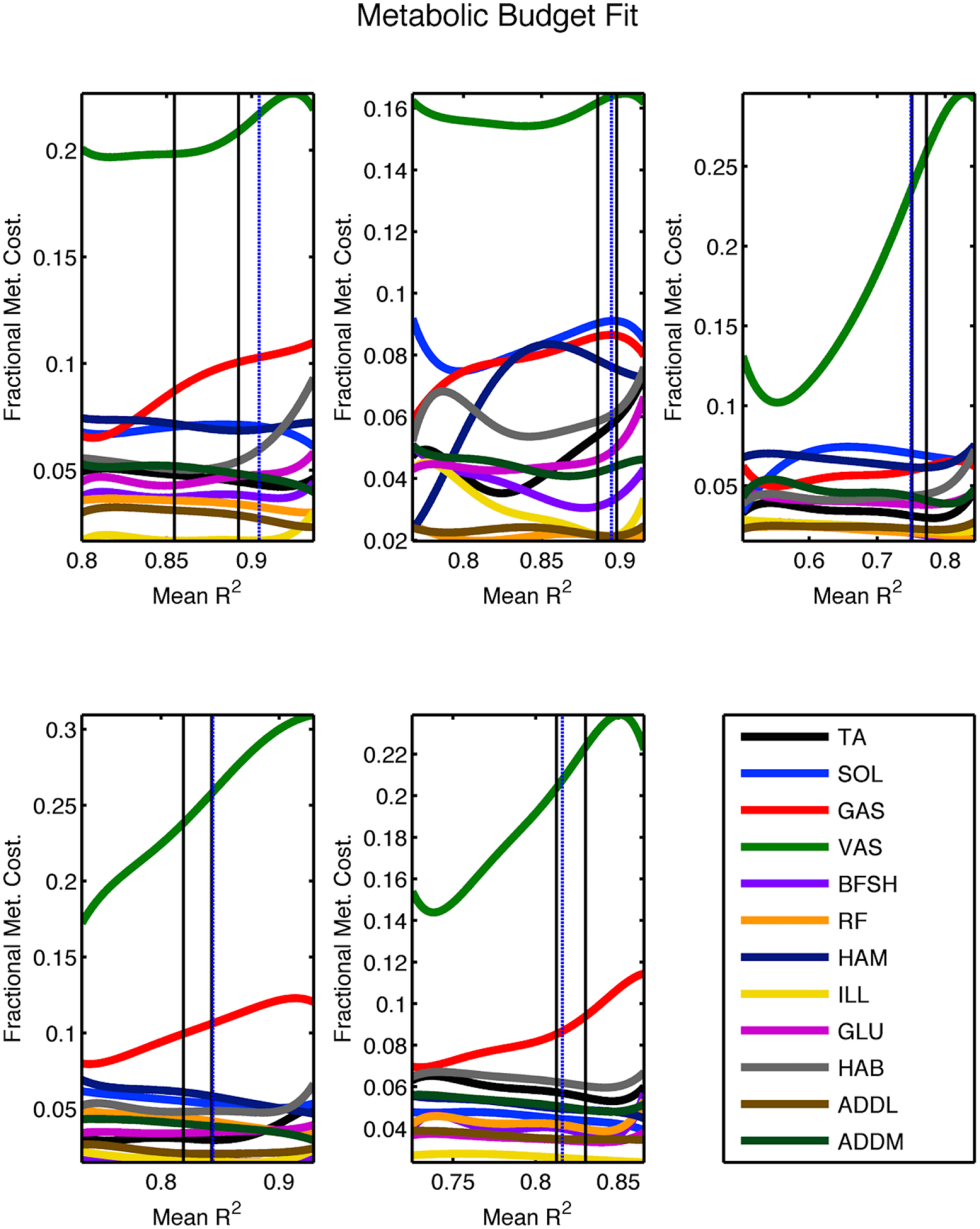
Fifth order polynomial fit to the per-muscle fractional metabolic cost as a function of kinetic fit along the Pareto Front, for each participant. The black lines are bounds on the observed metabolic cost while the dotted blue lines represent the locations of the chosen optimal solutions. These lines were derived by mapping the *R*^2^ values listed on the x-axis here to their corresponding metabolic costs in the solutions shown in Fig 4.

To generate predictions based on our model, we chose one optimal parameter set along the Pareto Front for each subject. This was accomplished by evaluating the per-muscle metabolic consumption among all Pareto optimal solutions (Fig 5). Within the set of solutions, those where the metabolic cost was low and the kinetic fit was poor were seen to have uniformly low expenditure per muscle. Those that had the very best kinetic fits but relatively large metabolic costs were seen to be sinking large amounts of metabolic energy into a small number of muscles to produce incremental improvements in the kinetic fit. Such a phenomenon was likely enabled by noise in the data (particularly in the EMG signals) and the imperfect ability of our lumped, partial muscle set to match the force produced by the full set of the human body. The ramp up in metabolic energy seen in this subset of muscles is not physical as it would lead to either rapid fatigue or to the muscle being modeled as much larger than it actually is (since muscle mass scales with *F*_*max*_). Hence we excluded solutions that displayed this behavior, choosing as optimal the remaining Pareto optimal solution with best kinetic fit. Mathematically this was achieved by setting a cutoff on the fractional expenditure of the vastus, as the metabolic cost of this muscle group was the largest and increased significantly as kinetic fit improved. The chosen fractional cutoff allowed us to apply one criterion to every participant to match the experimental metabolic cost, as shown in Figs 4 and 5. Table 1 shows how our choice of optimal solution is able to quantitatively match the experimentally-observed metabolic cost of transport in four out of five subjects (and on average) while maintaining acceptable joint moment agreement. The lone subject where quantitative metabolic agreement was not reached displayed only a 6% error. Further details of the cutoff are included in Materials and Methods and in S3 Text while the optimal parameter set for each participant is given at the end of this document.

**Table 1:** Performance of multi-objective optimization for chosen solution for each participant walking at self-selected speed. Experimentally measured and modeled metabolic cost of transport (MCOT) are shown as are the coefficients of determination for the modeled moments of the ankle, knee, and hip joints as compared to the data.

Participant	Experimental MCOT	Model MCOT	Ankle *R*^2^	Knee *R*^2^	Hip *R*^2^
1	0.32 ± 0.01	0.34	0.90	0.89	0.92
2	0.38 ± 0.01	0.38	0.86	0.89	0.94
3	0.35 ± 0.01	0.35	0.72	0.70	0.83
4	0.37 ± 0.01	0.37	0.82	0.79	0.92
5	0.34 ± 0.01	0.34	0.78	0.73	0.93
Mean	0.35 ± 0.02	0.36 ± 0.02	0.82 ± 0.07	0.80 ± 0.09	0.91 ± 0.04

To further evaluate the quality of our joint moment estimates we computed the fractional mean absolute error (FMAE)

$$\begin{array}{c} FMAE=\frac{1}{N}\frac{1}{\text{range}(\tau_{exp})}\sum_{i=1}^{N}\left|\tau_{exp,i}-\tau_{mod,i}\right| \end{array}$$ (2)

between the modeled joint moments *τ*_*mod*_ and the experimentally observed joint moments *τ*_*obs*_. These quantities are computed only over the stance phase to facilitate comparison with those generated from another current EMG-driven analysis, [31]. The quantities quoted from [31] represent an average over four different activities (walking, running, side stepping, and crossover) but are generally close to those they generate for walking only (except for the hip, which had lower errors for walking). Their analysis included two treatments; one that considered only one degree of freedom and one that considered multiple degrees of freedom (as in our model). In general our moment fits compare favorably (Table 2).

**Table 2:** Comparison of the fractional mean absolute error (FMAE) in our modeled joint moments and those generated by another current EMG-driven analysis [31]. Note that only the stance phase was considered here. The numbers provided from [31] represent an average over four different activities but are generally close to those they generate for walking only. Their analysis included two treatments; one that considered only one degree of freedom and one that considered multiple degrees of freedom (as in our model). In general our moment fits compare favorably.

Participant	Ankle FMAE	Knee FMAE	Hip FMAE
1	0.07	0.08	0.07
2	0.10	0.07	0.06
3	0.13	0.13	0.09
4	0.11	0.11	0.06
5	0.12	0.12	0.06
Mean	0.11 ± 0.02	0.10 ± 0.03	0.07 ± 0.01
Sartori et. al 1DOF	0.12 ± 0.03	0.12 ± 0.04	0.20 ± 0.04
Sartori et. al MDOF	0.09 ± 0.01	0.12 ± 0.05	0.20 ± 0.07

The metabolic expenditure of our model also aligns with previously published results. Across subjects, we found the average efficiency of positive muscle work to be 0.26 ± 0.02, consistent with [23, 32, 33]. We also estimated the metabolic expenditure of the model during different portions of the gait cycle by cross referencing the simulation with the input force plate data. The results are compiled in Table 3 and show a breakdown that is very similar to that simulated in [34].

**Table 3:** Fractional metabolic cost expenditure over the double support, single support, and swing phases of the gait cycle (as viewed from one leg). The average distribution for our five participants is seen to approximate that simulated in [34].

Participant	Double support	Single Support	Swing
1	.28	.50	.23
2	.23	.51	.26
3	.28	.46	.25
4	.27	.48	.26
5	.28	.41	.31
Mean	.27 ± .02	.47 ± .04	.26 ± 0.03
Umberger et al. 2010	.27	.44	.29

### Muscle Behavior in Optimal Solutions

**Fig 6:**
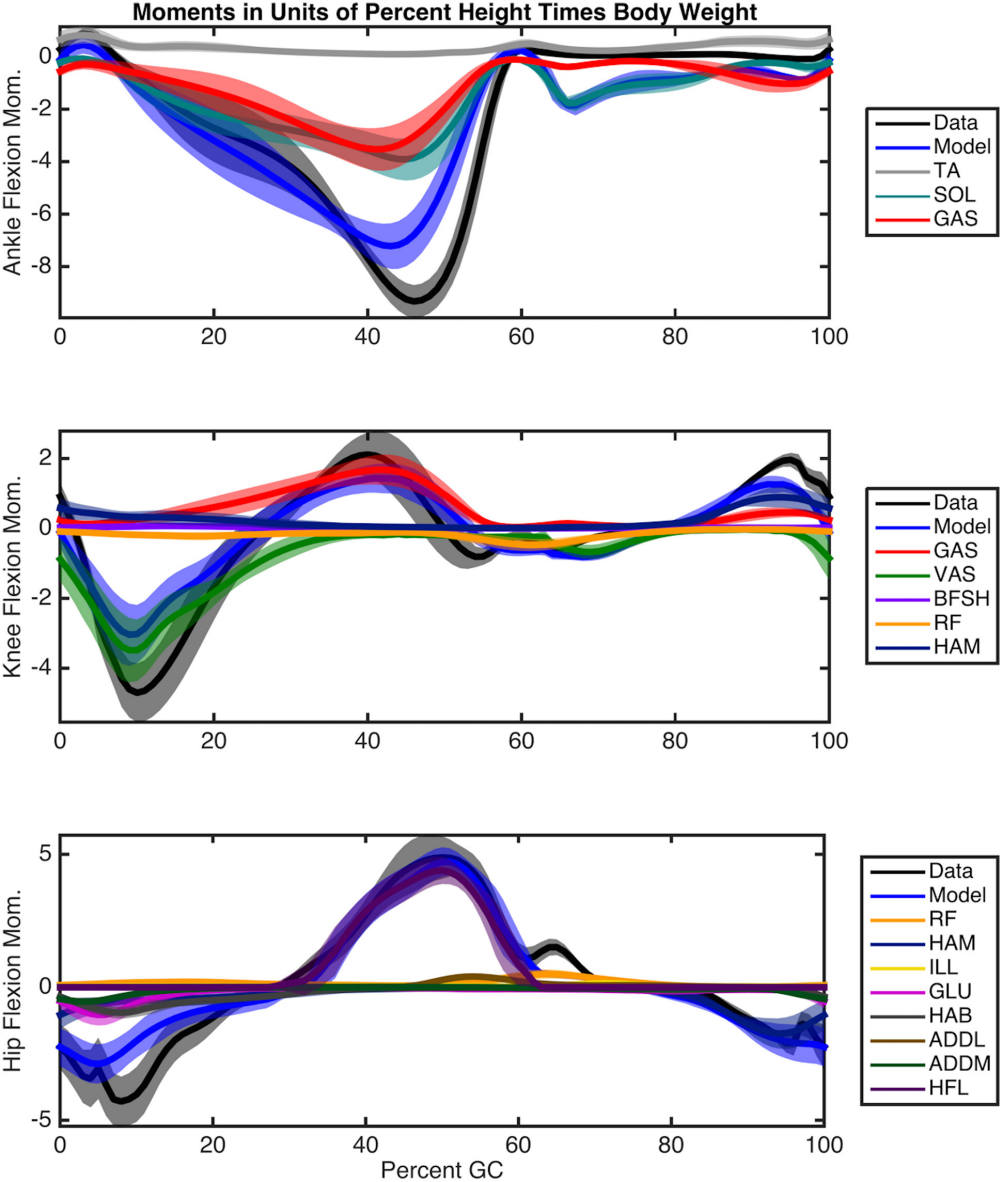
Contributions of individual muscles to joint torques, in terms of percentage of body weight times height and averaged over all participants. The error bands represent the standard deviation over different participants.

**Fig 7:**
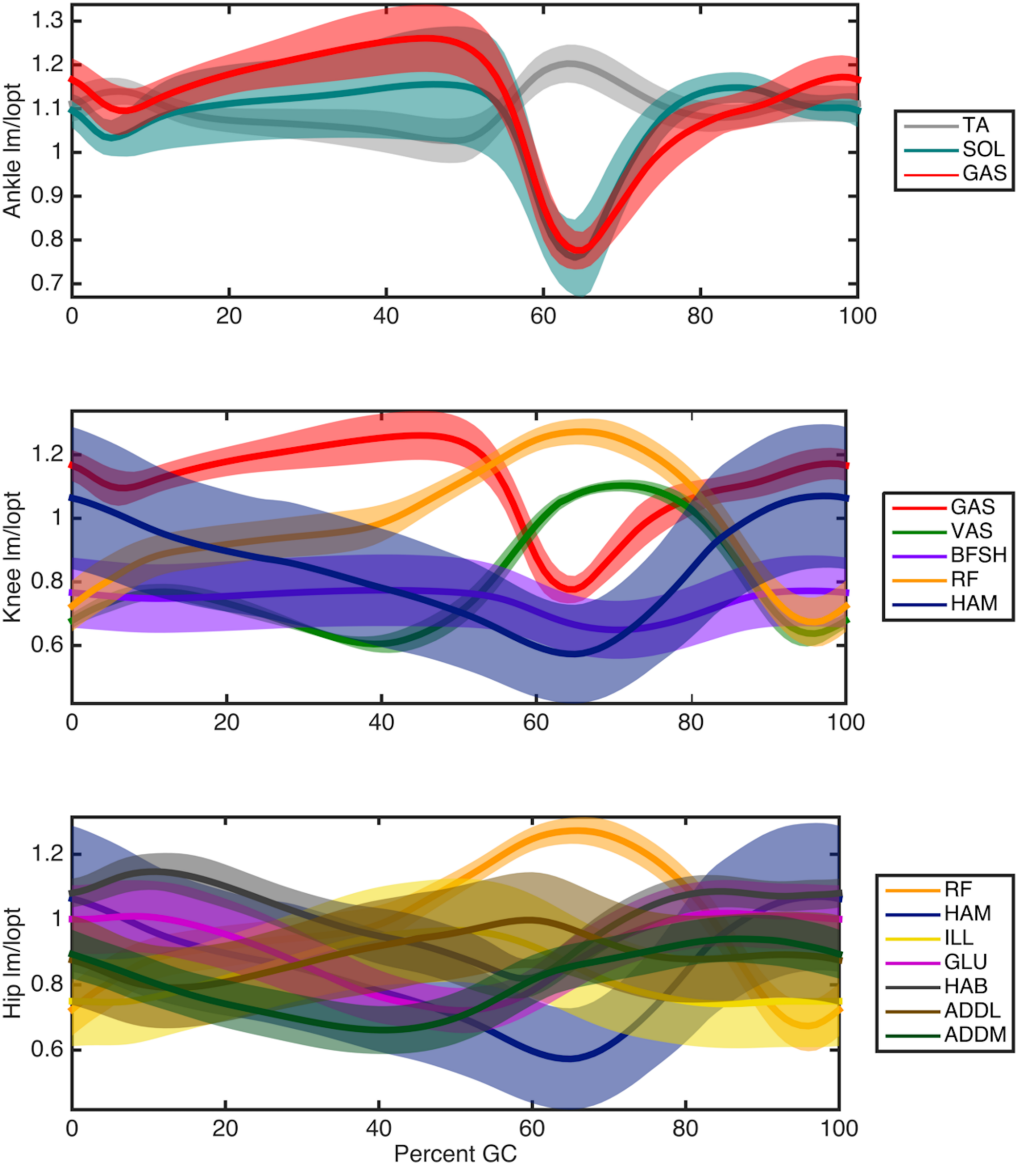
Muscle fascicle lengths produced by the model, normalized by optimal muscle length, and averaged over all subjects. The error bands of these profiles represent the standard deviation over different participants and their spread is largely provided by a constant offset rather than different shapes, as evidenced by the relatively constant standard deviations (over time) and Fig 8.

**Fig 8:**
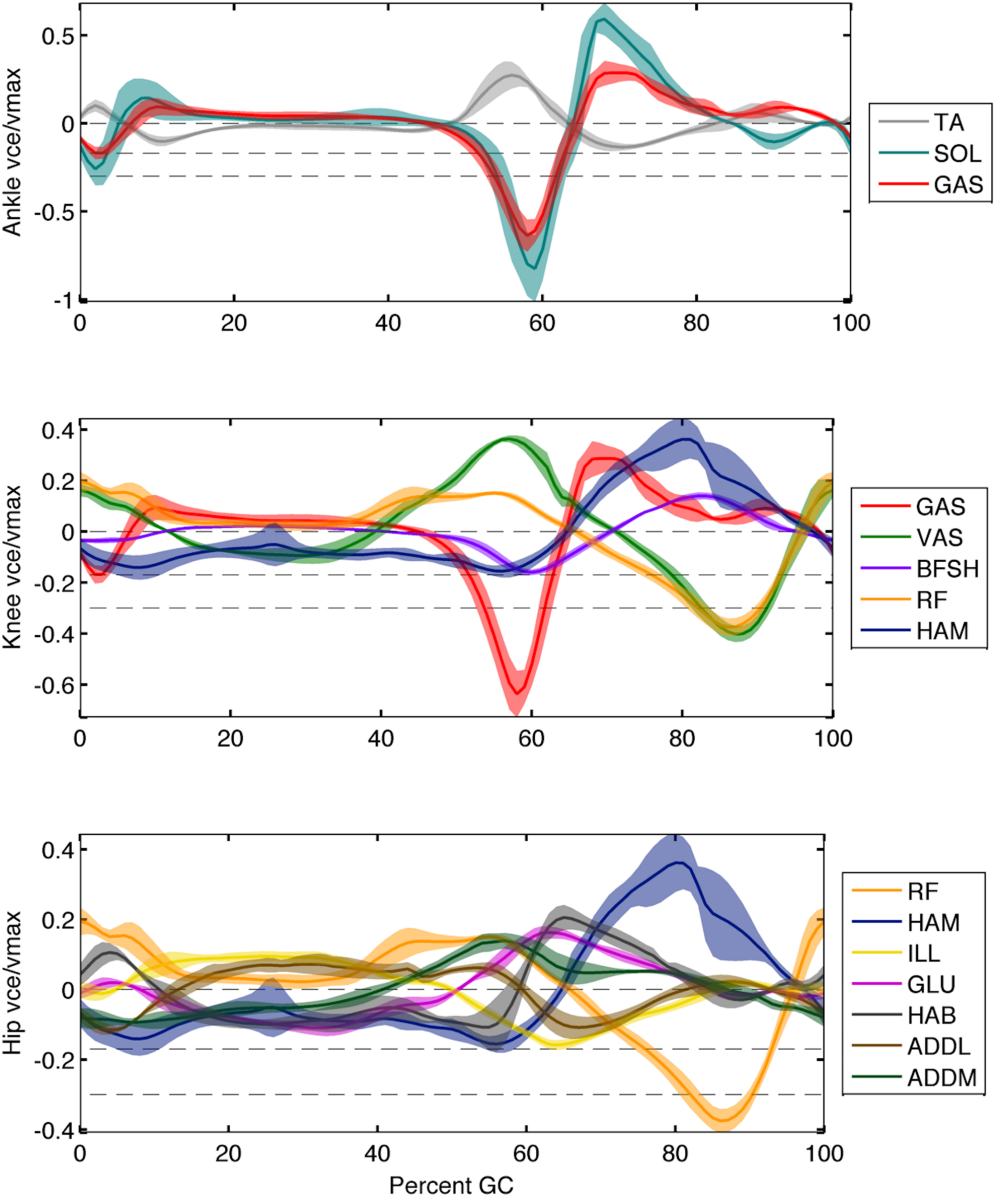
Muscle fascicle velocities produced by the model, normalized by maximum values (*v*_*max*_), and averaged over all participants. Here positive velocity refers to eccentric motion (muscle lengthening). The horizontal dotted lines at 0,−0.17,−0.30*v*_*ce*_/*v*_*max*_ represent the low energy, maximum efficiency, and maximum power operating speeds of muscle, respectively.

The optimal muscle-tendon parameter sets estimated by our optimization procedure provide a means to resolve the redundancy in joint actuation for each subject. When applied in conjunction with the computed kinematics and estimated muscle activations, individual muscle force and state may be estimated. Fig 6 shows the torque breakdown for each joint in terms of percentage of body weight times height and averaged over all subjects. Fig 7 shows the trajectories of the muscle fascicle length normalized by *l*_*opt*_ and averaged over all subjects. Note that the spread of some of these profiles is not due to a difference in general shape but rather to an overall offset in length, as evidenced by their nearly constant standard deviations. The soleus, hamstring, and vastus fascicle lengths in this plot all agree quite well in both shape and offset with the predictions in [35]. The soleus force and length also agree qualitatively with the projections of [11]. Fig 8 shows the velocity trajectories for each muscle fascicle normalized by its maximal value *v*_*max*_ and again averaged over all participants. As can be seen from the small variation in the shapes of the profiles in these plots, muscle force and state followed similar trajectories across participants. These predictions were also seen to vary little along the Pareto front in the experimentally measured metabolic band of a given subject.

**Fig 9:**
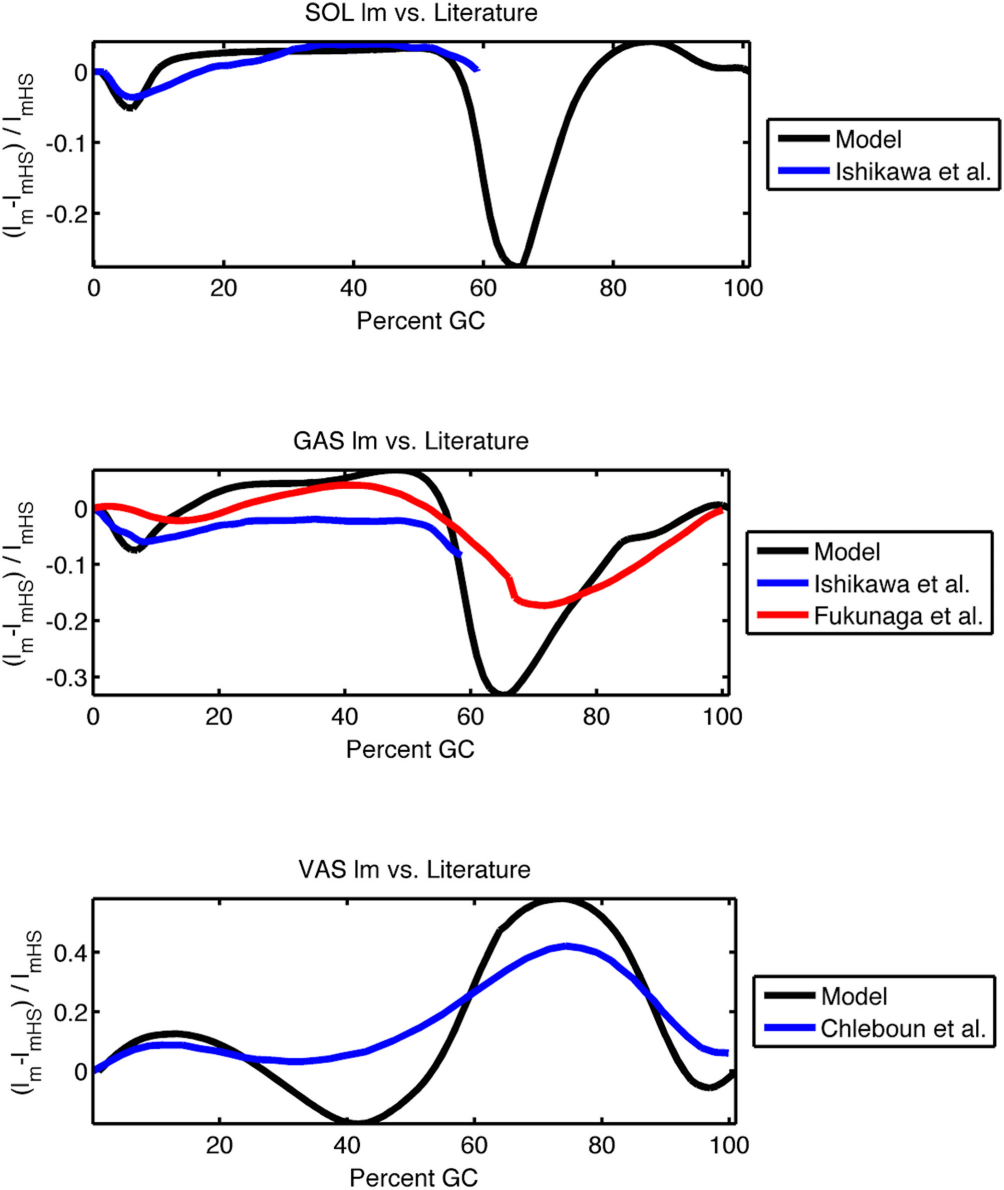
Comparison of model fascicle trajectories with *in vivo* ultrasound data [7, 8, 10]. In each case the muscle fascicle lengths *l*_*m*_ are normalized by their length at heel strike, *l*_*mHS*_. In our study the modeled muscle was taken from the subject who most closely matched the average height and weight of the experimental study.

In general, muscle fascicle state is extremely difficult to measure. The only currently available means to obtain these profiles is ultrasonography, which is only practical for the relatively short distal muscles of the leg. In Fig 9 the modeled fascicle trajectories of muscles from one subject are compared with the experimental profiles available from published ultrasound studies. In each case the muscle fascicle lengths *l*_*m*_ are normalized by their length at heel strike, *l*_*mHS*_. To generate these plots we took the modeled muscle from the subject who most closely matched the average height and weight of the experimental study. Soleus and gastrocnemius profiles came from Ishikawa et al [8], a gastrocnemius profile came from Fukunaga et al [7], and the vastus lateralis profile came from Chleboun [10]. In the plantar flexors, long stretches of nearly isometric operation are observed in mid-stance in both the model and *in vivo* profiles. In the vastus, the fascicle trajectory is seen to somewhat track the flexion of the knee in both the model and the published data. However while the qualitative trends of each muscle are consistent, quantitative agreement is not observed. We believe that observed differences come from (i) the difference in walking speed between our study and the literature, (ii) natural variation in the kinematics of early stance (which affects the initial muscle length for normalization), and (iii) uncertainty in the breakdown of what constitutes muscle and what constitutes tendon in ultrasound studies. We hope that future experimental methodologies, perhaps employing implantable sensors, will be able to further test our predicted fascicle trajectories.

## Discussion

Several observations may be made about both the methodology employed and the results obtained in this study. On the methodology side, we first address the steps taken to estimate the activations of the muscles in our model. As mentioned above, the conventional approach for estimating muscle excitation based on EMG data involves a bandpass filter [4–6]. We tried this approach with our optimization scheme (on all subjects) but found that it provided an inferior ability to match the observed joint moments compared to the implemented method. Digging further into this we found that two main differences existed between the approaches; (i) the chosen Bayesian method produces a profile that turns on and off more sharply than the signal produced by the bandpass filter and (ii) the signal produced by the Bayesian method consistently lags the bandpassed signal by about 50 ms. The sharpness of the profile does not affect the results significantly as it is mostly washed out by the ensuing activation dynamics and averaging. However the time dependence does matter; this lag enables the build up of muscle force in a manner consistent with the observed joint torques. Interestingly we found that [36] introduced a 40 ms lag to their EMG signal to “account for electromechanical delay between surface EMG and force production.” This lag was employed by other studies [37, 38] and fell within the 10−100 ms range given for these processes in the literature [39, 40]. We found that adding a 40 ms lag as in [36] to the excitations produced by bandpassed filtered EMG signals gave performance nearly as good as those provided by the Sanger algorithm, with less variation among gait cycles. However this lagged bandpass method does not lend itself to a biophysical interpretation as clearly as the Bayesian model does.

Despite our best efforts at EMG data collection and activation estimation, deficiencies in this part of our data set clearly exist. Surface EMG in general is prone to noise and artifacts, and Sanger’s algorithm (tested only isometrically in his publication [25]) does not remove them. While the effects of inconsistent artifacts was minimized by discarding the EMG data from clearly compromised gait cycles and the use of average trajectories in our model, they were likely not removed entirely. Better results may be obtained in future work through fine wire EMG measurements, which while more invasive are known to produce more reliable signals. Here generic fine wire EMG profiles reported in [28] were used to replace the noisy surface EMG measurements of the muscles spanning the hip. Interestingly our model actually displayed slightly better agreement with the observed hip torque profiles than with those of the other joints, but this is misleading as most of the modeled hip moment came from the hamstrings and the hip flexor ligament (which were unaffected by the generic profiles).

A more minor deficiency in the EMG pipeline was normalization by the maximal voluntary contraction (MVC) values. While we do not know of a better alternative, this approach did lead to the normalized excitations of our muscles occasionally exceeding one in fast walking trials. When that occurred we renormalized by the value in the fast walking trial and reprocessed, but the normalization constant could still have been too small in other cases. Fortunately the impact of this scaling is extremely minimal because the estimated activation directly multiplies the maximum isometric force *F*_*max*_, which is optimized. A small effect remains because the normalization occurs before the excitation undergoes the activation dynamics (5), but that effect is largely irrelevant to our results.

The system identification component of this study produced a methodology for estimating muscle-tendon parameters capable of matching the measured metabolic consumption while producing joint torque profiles that tracked observations reasonably well. One criterion based on the metabolic consumption of the vastus muscle group (the largest in the model) enabled the metabolic match for all participants. While the accuracies of our modeled joint torque profiles compare favorably with other current EMG driven modeling procedures [31], they do tend to underestimate the required joint torques. This characteristic is likely due to the exclusion of some muscles as well as the lumping of some muscle groups. In particular the deficiency exhibited in ankle moment during late stance is likely due to the exclusion of smaller plantar flexor muscles which are known to engage during that time [28]. Similarly the lumping of the three hamstring muscles (semimembranosus, semitendinosus, and biceps femoris long head) may be responsible for some of the deficiencies observed in the knee flexion moments throughout the gait cycle. Further resolution of these and other muscle groups where the muscle activations are not quite concurrent and the muscle-tendon lines of action are not quite aligned would likely improve the predictive power of this approach, allowing more accurate determination of the roles of each muscle during a given task.

One notable aspect of the model was the importance of the hip flexor ligament, which produced nearly all of the required hip flexion moment near toe off at no metabolic cost. Its linear form was chosen as in [29] for maximal simplicity, but did not agree with the nonlinear damped form used in previous work [18, 41]. Given that it produced torque for free it may have suppressed the required action of the other hip flexors (notably the iliacus) near toe off and produced a better hip moment fit than would have otherwise been possible for the same metabolic cost. However the suppression of other hip flexors could not have been large as these muscles are not strong enough to produce the required torque alone and are not significantly stretched in this time frame. It is also known that the hip ligaments produce the flexion torque necessary to balance the upper body against gravity in a standing position, where the line of gravity passes posterior to the hip joint [42]. They enable people to stand erect and even carry extra weight without significant muscle work at the hip, allowing a low metabolic cost to be maintained [43]. Since the engagement angles we used are consistent with standing and damping in human connective tissue is believed to yield only a slight drift over walking time scales [16], we believe that the contribution of the hip flexor ligament in our model is physiologically reasonable.

The optimal muscle-tendon parameters found in this study play different roles in facilitating efficient locomotion. The maximal muscle isometric force (*F*_*max*_) must be large enough to meet the torque requirements at each joint, but small enough to keep muscle size and metabolic cost reasonable. The tendon slack lengths *l*_*sl*_ and muscle optimal lengths *l*_*opt*_ govern the timing of force production, acting in concert with muscle activation. The tendon shape parameters λ_*ref*_ and *K*_*sh*_ define the elastic properties of the tendon and are tuned to ensure correct muscle operation and joint actuation. The resulting muscles and tendons together produce an interconnected system capable of producing the joint torque necessary for locomotion at a minimal metabolic cost.

Evaluating the velocities at which the muscles in our model contract while activated can lend insight to the goals of their control. As was emphasized in [29, 44], muscles minimize metabolic consumption at low speeds (i.e. when operating approximately isometrically). Further, A.V. Hill [45] demonstrated that skeletal muscle maximizes its efficiency while shortening at *v*_*CE*_ ≈ −0.17*v*_*max*_ and its power output at *v*_*CE*_ ≈ −0.30*v*_*max*_. These three speeds (*v*_*CE*_ = 0,−0.17,−0.30*v*_*max*_) are indicated by the horizontal dashed lines in Fig 8. Combining this with Fig 3 allows us to contextualize the modeled velocities of each muscle when activated. At the ankle, the tibialis anterior is seen to operate at low speeds when engaged. This minimizes metabolic cost and is consistent with isometric contractions, as modeled in [29, 44]. Both the soleus and gastrocnemius are seen to operate approximately isometrically through their activation in mid-stance before rapidly increasing their contraction velocity in a power stroke toward the end of stance (≈ 60% GC). The required positive work of the plantar flexors is consistent with the need seen in [29, 44], but their rapid contraction at the end of stance does not strictly agree with the efficiency goal of the soleus and power goal of the gastrocnemius noted in [23]. This inconsistency amounts to a phase difference; the observation in [23] was based on muscle speed at toe off but our plantar flexor velocities reach approximately the same levels around 53% GC. At the knee, most muscles are seen to operate approximately isometrically. This agrees with [29, 44], which note that these muscles primarily serve to modulate the stiffness of the joint in an optimally economical fashion. It is worth noting that the vastus group is seen to re-engage at the end of swing near the the optimal efficiency regime (extending the knee for heel strike) before returning to the low speed regime. At the hip, most muscles are observed to contract at low speed when activated. Two exceptions are the iliacus and the adductor longus, both of which gravitate toward maximal efficiency as they flex the hip around toe off. The adductor magnus may also approach the maximal efficiency regime as it extends the hip around heel strike, but this is less clear. Endo et al [29, 44] found that the muscles spanning the hip could not be modeled strictly isometrically, consistent with these observations.

Several avenues exist for extending this work in future studies. One route would be to expand the model past the sagittal plane; as shown in [31] matching moments in three dimensions could have an impact on the optimized parameter sets. Another route would be to validate the model by testing under different walking conditions. If more reliable EMG measurements could be obtained, we could train the model based on level-ground walking at self-selected speed and then evaluate the ability of the optimal parameter set to match the observed joint torque profiles and metabolic costs under different conditions (speeds, inclines, etc.) In this case new experimental observations of the change in plantar flexor function across speed [12, 13] could be used to evaluate the model. Another possibility is that our overarching hypothesis is incomplete- maybe the body, instead of adapting its morphology to maximize its efficiency at (the presumably most common task of) walking at self-selected speed, actually optimizes with multiple tasks in mind. One could imagine additionally collecting data from a subject running a self-selected speed and adding extra dimensions to the cost function wherein that task is optimized concurrently with walking at self-selected speed. The procedure followed here for self-selected speed walking could thus become part of a larger optimization scheme by including other tasks.

While such experiments could lend considerable insight, they will undoubtedly prove challenging due to the difficulties associated with collecting EMG under the stated conditions. One alternative would be to simulate the neural control of the model. Recent forward dynamic models driven by reflexive feedback have shown the ability to walk stably across different terrains [19, 46], at different speeds [47, 48], and in three dimensions [48, 49]. These models are built for a subject with “average” dimensions and could therefore be improved through data-driven customization for individual subjects and the inclusion of more realistic muscle-tendon geometries and morphologies. This would include scaling segment inertias, refining muscle-tendon lines of action and moment arms [14], and optimization of muscle-tendon morphologies to simultaneously minimize metabolic cost and maximize agreement to experimentally observed kinematic and/or kinetic data. In this way a more realistic representation of an individual subject could be obtained, leading to further insights about the roles of individual muscles and tendons during gait and their variation amongst subjects. One could also imagine altering the neural control in this paradigm and using the framework to study movement disorders such as cerebral palsy.

## Materials and Methods

### Ethics Statement

The experiments of this study were conducted in compliance with the principles of the Declaration of Helsinki. The study was approved by the MIT Committee on the Use of Humans as Experimental Subjects (Protocol 1101004266). Prior to the experiments all participants provided written consent for data collection, analysis, and publication.

### Data Collection

Kinematic, kinetic, electromyographic, and metabolic data were collected at the Harvard University Skeletal Biology Lab. Five healthy adult males participated in the study, with their average height, mass, and age being 1.77 ± 5 m, 70.4 ± 6.3 kg, and 26 ± 2 years, respectively. The required data sets were collected in two phases. First the subjects were outfitted with a portable oxygen consumption mask attached to a Cosmed *K*4*B*^2^ *VO*_2_ system. This system employs a standard open-circuit gas analysis technique to estimate metabolic energy consumption based on measurements of oxygen inspired and expired [50]. Three of the subjects were asked to stand still for seven minutes while a basal measurement was recorded, with the rates of the other two participants taken to be the mean of these three closely grouped rates (1.38 ± 0.06 W/kg, 1.60 ± 0.07 W/kg, 1.63 ± 0.06 W/kg). Participants then walked barefoot on an instrumented treadmill for seven minutes at each of six speeds (0.75 m/s, 1.00 m/s, 1.25 m/s, 1.50 m/s, 1.75 m/s, and 2.00 m/s), allowing the variation of metabolic energy expenditure to be measured across speed. The results were quickly tabulated and used to estimate the walking speed where the metabolic cost of transport (MCOT) was minimal.

Once the metabolic cost measurements were completed, the oxygen consumption mask and Cosmed system were removed and each participant was outfitted for the second phase. In this phase kinematic, kinetic, and electromyographic data were collected for two minutes of barefoot walking at each of seven speeds; the six listed above and the speed where the subject’s MCOT was found to be minimal. An infrared camera system (8 cameras, Qualisys Motion Capture Systems, Gothenburg, Sweden) was used to track the motion of subjects as they walked in the capture volume. Reflective markers were placed at 43 (bilateral) locations on the participant’s body and their three dimensional trajectories were recorded at 500 Hz. The marker locations were chosen specifically to track joint motion, as prescribed by the Helen Hayes marker model. The ground reaction forces and contact centers of pressure were measured using a split-belt instrumented force plate treadmill (Bertec Corporation, Columbus, OH). Electromyographic signals were collected using a surface system from Motion Lab Systems (Baton Rouge, LA) and electrode placements as dictated in [51]. Fourteen muscles (tibialis anterior, soleus, medial gastrocnemius, vastus lateralis, biceps femoris shorthead, rectus femoris, semimembranosus, biceps femoris long head, illiacus, gluteus maximus (lower), gluteus maximus (upper), gluteus medius, adductor longus, and adductor magnus) on one leg of each subject were recorded, with symmetry being assumed for the other leg. The signals were recorded at the surface using pre-gelled bipolar electrodes (Electrode Store Model BS-24SAF, part number DDN-20), sampled at 1000 Hz, and amplified 20 times by pre-amplifiers (Motion Lab Systems, part number MA-411). Prior to walking trials a maximum voluntary contraction (MVC) trial was conducted for each muscle group wherein the participant was asked to work that particular group as hard as possible. These MVC trials were used for normalization purposes in muscle excitation estimates.

#### Estimation of joint dynamics and muscle-tendon geometries

The marker and force plate data were downsampled to 125 Hz and processed using SIMM (Software for Interactive Musculoskeletal Modeling, Musculographics Inc., Evanston, IL). A static trial was used to scale the SIMM full body dynamic model for each participant, with all subsequent analyses being based on that scaling. Joint angles, muscle-tendon lengths, and muscle-tendon moment arms for each trial were obtained through an inverse kinematic analysis. Joint moments were computed using the SIMM Dynamics Pipeline, which utilizes the SDFAST Software (PTC, Needham, MA). All resulting trajectories were broken into gait cycles, normalized temporally to percent gait cycle, and averaged. Gross outliers (typically caused by gait irregularities or lost markers; on the order of 5% of gait cycles) were removed prior to averaging.

#### Muscle activation estimation

Muscle activation provides a measure of a muscle’s active force generation capability as a function of time. It is defined as the relative amount of calcium bound to troponin in a muscle, with higher values indicating more potential for cross bridge formation and higher muscle forces. Activation is driven by electrical neural excitation signals and may therefore be estimated through EMG measurements. Surface EMG signals represent a combination of the action potentials, depolarization currents, and ion flows within a muscle. They provide valuable information for resolving muscle contributions but, due to large signal variability and measurement artifacts, must be carefully processed to provide reliable inputs to quantitative models.

Standard EMG analysis models the signal as an amplitude-modulated band-limited noise source [4], rectifying and low pass filtering it to produce an amplitude envelope [5, 6]. While elegant, this approach did not perform as well in our optimization problem as a more recently introduced method by Sanger [25]. As detailed above, we found that the slight delay in the profiles produced by Sanger’s method (relative to bandpass-based methods) allows our model to more naturally build up the required muscle force. Sanger’s method models the EMG signal as a filtered random process with random rate and seeks to infer an underlying neural driving signal *x*(*t*) that evolves as

$$\begin{array}{c} dx=\alpha \left(dW\right)+\left(U-x\right)dN_{\beta }. \end{array}$$ (3)

In this continuous-time stochastic differential equation *dW* is the differential of standard Brownian motion with rate *α*, *dN*_*β*_ is the differential of a counting process with *β* events occurring per unit time (the jump term), and *U* is a uniformly distributed random variable in [0, 1]. Without the driving *dN*_*β*_ term this equation would amount to a random walk. The hidden state *x*(*t*) is measured via

$$\begin{array}{c} P\left(\text{emg}|x\right)=\frac{\text{exp}(-\text{emg}/x)}{x}, \end{array}$$ (4)

where *P*(emg|*x*) is the conditional probability of observing a rectified raw signal emg given driving signal *x*(*t*). This model reflects the observed Laplacian distribution of relevant EMG signals and wraps all elements of the measurement- the various fibers and electrical sources, the filter of skin and fat, the placement and impedances of the electrodes- into one equation. Combining these relations with Bayes’ Rule and solving recursively, Sanger derives the maximum a posteriori (MAP) estimate of the driving signal, i.e. the *x*(*t*) that maximizes *P*[*x*(*t*)|emg(*t*), emg(*t*−1),..].

The resulting driving signal has been shown to accurately track the turn on and turn off times of torque profiles produced by maximal isometric contractions while minimizing the noise floor when a muscle is inactive [25]. However, as noted in [23], it cannot correspond to expected activation signals because (i) the near instantaneous transitions are too sharp and (ii) the profiles do not build up while activated. These differences stem from the disparity between the modeled jump-diffusion process and the true build up of muscle activation. The sudden jumps in [25] capture the start and stop of neural excitation signals, but do not account for the time required for the slower calcium binding dynamics. Further, the muscle active state is known to rise faster than it decays [52]. This feature is not present in the Sanger model; the diffusion and jump rates are symmetric with respect to the derivative of *x*(*t*). Combining these facts it becomes clear that the hidden state *x*(*t*) more closely represents the neural excitation of the muscle than the muscle active state. To obtain an activation estimate, we apply activation dynamics in the form [26, 27]

$$\begin{array}{c} \dot{a}=\left\{\begin{array}{cc} \left(x-a\right)\left[x/\tau_{\text{act}}+\left(1-x\right)/\tau_{\text{deact}}\right], & x\geq a\\ \left(x-a\right)/\tau_{\text{deact}}, & x<a \end{array}\right. \end{array}$$ (5)

to the output of the Sanger algorithm *x*(*t*). Here *a* is muscle activation while *τ*_*act*_ and *τ*_*deact*_ are the activation and deactivation time constants, respectively. These time constants are known to depend on muscle fiber composition; the specific values used for each muscle in this study are given in S2 Text. The resulting estimate combines the timing inferred by the Sanger algorithm with the known biophysics of muscle activation.

The complete approach was implemented on collected EMG data using MATLAB (Mathworks, Natick, MA). The raw signal of each EMG data channel was preprocessed by removing the DC offset, clipping the signal beyond 5 standard deviations, normalizing to the resulting maximum value, and rectifying. The clipping was first included by Sanger to prevent the Bayesian algorithm from trying to estimate conditional densities from exceedingly rare values, which cannot be done accurately and likely result from artifacts. The data was then passed through the Sanger Bayesian algorithm described above to estimate the neural excitation *x*(*t*) of each muscle (link to source code in References). The diffusion and jump terms (*α* and *β*) in [25] were adjusted to 0.5 and 5 × 10^−31^, respectively, to allow weaker/shorter signals to more reliably be captured without introducing excess random jumps in the output signal or affecting its timing. The drawback of this tuning was a decrease in sharpness of the turn on/turn off of muscle excitations, but this would have occurred in the ensuing filtering and averaging anyway. The resulting excitations were re-normalized by values estimated during maximal voluntary contraction (MVC) trials. The MVC trials were processed in the same way with the average magnitude of the largest burst (of duration at least 1 s) being taken as the MVC value. Occasionally we found that an MVC trial was exceeded in fast walking trials; in that case we renormalized to the larger value and reprocessed. The normalized profiles were then thresholded to remove the noise floor and passed to the shaping filter (5) representing muscle activation dynamics, which was implemented using the ode15s (stiff/NDF) solver in MATLAB Simulink. The output activation profiles *a*(*t*) were broken into gait cycles, normalized temporally to percent gait cycle, and averaged. Gross outliers (about 10% of gait cycles) were again discarded; in this case additional error sources included motion artifacts and instances of poor electrode connection. The resulting average activation estimates are plotted at the speed of minimal MCOT for all subjects in Fig 3.

Unfortunately not all EMG channels provided satisfactory activation estimates, with low signal to noise ratios being observed particularly in muscles spanning the hip. This was caused by the relatively complicated geometry of that joint, the preponderance of associated motion artifacts, the muscles being located deep beneath the skin, and the relative lack of access to that area. Such problems can be minimized through the use of fine wire electrodes, as in [28], and in fact we found that the profiles in [28] could be used to more precisely estimate the turn on and turn off times of the neural excitation signals for the muscles spanning the hip than our surface measurements. Hence we used these literature profiles to produce neural excitation profiles for the iliacus, adductor longus, adductor magnus, gluteus maximus, and gluteus medius. The profiles were digitized using MATLAB, specifically the “ginput” function. The literature profiles were given in term of percent gait cycle; for each participant we resampled to make the duration of the profile match that of the subject’s average gait cycle. No amplitude renormalization was necessary. The resulting profiles were then passed through the dynamics (5) to produce activation estimates. In the infrequent event that the data from another muscle was not salvageable for a given subject, the trajectory of that muscle was taken to be the average of the trajectories for that muscle in all subjects where the measurement was acceptable. Further details and a statistical analysis of this approach are given in S1 Text.

### Musculoskeletal Model

The processed data were used to build a dynamic model of the leg during walking (Fig 1). The model includes all muscles that make significant contributions to the components of ankle, knee, and hip torque perpendicular to the sagittal plane during walking. Several muscle groups were lumped together for simplicity; this was deemed appropriate if all muscles within a group had similar lines of action and were activated simultaneously during walking. These included the GAS group (medial and lateral gastrocnemius), the VAS group (vastus lateralis, medialis, and intermedius), the HAM group (semimembranosus, semitendinosus, and biceps femoris long head), and the ILL group (iliacus and psoas). Each muscle group had one effective tendon to represent the net compliance of the muscle’s interaction with the skeleton. In addition to the muscle-tendon units a passive ligament spanning the hip was included to allow for the recovery of elastic energy in that joint. This element represents the contributions of the iliofemoral, ischiofemoral, and pubofemoral ligaments as well as those of other connective tissue at the hip and was modeled using a nonlinear equation in [18]; for simplicity we took it to be a linear spring that only engages around the time the hip goes vertical and begins to extend [29]:

$$\begin{array}{c} \tau_{HFL}=-K_{HFL}\left(\theta_{hip}-\theta_{0,HFL}\right). \end{array}$$ (6)

Note that both the moment and the angle in this equation were defined with flexion being positive. Below we describe the muscle, tendon, and joint dynamics of the system.

#### Muscle dynamics

All muscles in the model were taken to have Hill-type contraction dynamics similar to those described in [19]. Each muscle contains a contractile element (CE) that represents the active muscle fibers and a parallel elastic element (PE) that represents the elastic structures surrounding the muscle. The contractile element force *F*_*CE*_ depends on the muscle activation *a*, the contractile element length *l*_*CE*_, and the contractile element velocity *v*_*CE*_. The parallel elastic element force *F*_*PE*_ depends only on the contractile element length and engages only above the optimal fiber length *l*_*opt*_. *l*_*opt*_ and the maximal isometric force *F*_*max*_ vary with muscle size while the maximal contractile element velocity *v*_*max*_ varies with muscle fiber composition. For further details on the implemented muscle model including predefined constants see S2 Text; for details on how the differential equations were solved see [19] and its accompanying source code.

#### Tendon dynamics

As mentioned above, tendons are non-linear elastic elements that join muscle to bone. Their force-strain relation may be modeled by the general form [16]:

$$\begin{array}{c} F_{SE}\left(\lambda \right)=\left\{\begin{array}{cc} F_{max}\frac{\text{exp}\left(\frac{K_{sh}}{\lambda_{ref}}\lambda \right)-1}{\text{exp}(K_{sh})-1}, & \lambda >0\\ 0, & \lambda \leq 0, \end{array}\right. \end{array}$$ (7)

where

$$\begin{array}{c} \lambda =\frac{l_{SE}-l_{sl}}{l_{sl}} \end{array}$$ (8)

is the strain of the tendon beyond its slack length *l*_*sl*_. Here *F*_*max*_ is the maximum muscle isometric force, *K*_*sh*_ is a shape factor, and λ_*ref*_ is a reference strain. *K*_*sh*_ determines where the force-length curve transitions from its flat lower (“toe”) region to its nearly linear behavior for large strains. λ_*ref*_ is the strain where *F*_*SE*_ = *F*_*max*_. These four parameters define the morphology of a particular tendon; *F*_*max*_ and *l*_*sl*_ scale with the size of the tendon while *K*_*sh*_ and λ_*ref*_ depend on the material properties of the tendon.

#### Muscle-tendon dynamics

Muscle and tendon act in series but are typically oriented obliquely, with the angle in between the two being known as the pennation angle. The forces exerted by the tendon (SE), muscle (M), and full muscle tendon complex are:

$$\begin{array}{c} F_{MTC}\left(t\right)=F_{SE}\left(t\right)=F_{M}\left(a\left(t\right),l_{CE}\left(t\right),{\dot{l}}_{CE}\left(t\right)\right)\text{cos}\left(\theta \left(t\right)\right). \end{array}$$ (9)

Here the muscle contractile element length *l*_*CE*_ represents the state variable. The total length of the muscle tendon complex is

$$\begin{array}{c} l_{MTC}\left(t\right)=l_{SE}\left(t\right)+l_{CE}\left(t\right)\text{cos}\left(\theta \left(t\right)\right). \end{array}$$ (10)

The pennation angle *θ*(*t*) varies so as to keep the width of the muscle approximately constant [15] and can be written as a function of fascicle length:

$$\begin{array}{c} \theta \left(l_{CE}\right)={\text{sin}}^{-1}\left(\frac{l_{opt}\text{sin}\left(\theta_{0}\right)}{l_{CE}}\right). \end{array}$$ (11)

Here the angle *θ*_0_ is the pennation angle when *l*_*CE*_ = *l*_*opt*_ (which is very near the resting length of the muscle [16]). It was found that allowing this angle to vary with fascicle length was necessary to obtain muscle fascicle trajectories that produced human-like metabolic cost estimates. The total muscle torque at a given joint is then the sum of all muscle-tendon unit forces multiplied by their respective time-varying moment arms *r*_*i*_(*t*):

$$\begin{array}{c} \tau_{mod}=\sum_{i}F_{MTC,i}\left(t\right)r_{i}\left(t\right). \end{array}$$ (12)

To resolve the redundancy in joint actuation, each time varying muscle-tendon force *F*_*MTC*,*i*_(*t*) must be determined.

### Optimization Inputs, Outputs, and Parameters

An overview of the applied optimization procedure is shown in Fig 2. There were two categories of inputs for each muscle in our model: (i) *a*(*t*), *l*_*mtc*_(*t*), and *r*_*i*_(*t*)- all estimated from the data and (ii) muscle-tendon morphological parameters ${\vec{m}}_{i}$ which were identified via optimization. Morphological parameters which affect the Hill-type contraction dynamics of the modeled muscles include muscle maximum isometric force *F*_*max*_, length where active muscle force is maximal *l*_*opt*_, fiber composition *FT*, tendon slack length *l*_*sl*_, tendon shape factor *K*_*sh*_, and tendon reference strain λ_*ref*_. We evaluated the consequences of varying all of these variables, finding differing levels of sensitivity for each. The variables to which the model was insensitive were fixed, leaving *F*_*max*_, *K*_*sh*_, λ_*ref*_, and an overall scaling factor for *l*_*sl*_ and *l*_*opt*_ as the parameters to optimize. The last factor was chosen to ensure that the muscle operated in reasonable regions of its force-length space while preventing overfitting. The ratio *l*_*sl*_/*l*_*opt*_ is known to vary significantly among muscles but not significantly in the same muscle among subjects [14], justifying this choice.

As mentioned above, our optimization is built on the hypothesis that the muscle-tendon morphologies of the muscles comprising the leg have evolved to minimize the metabolic cost of walking at the speed where the MCOT is minimal. This assumption is supported by the fundamental importance of bipedal walking as a means of transport and the tendency of humans to walk at or near this speed. For each subject we therefore sought a set of morphological parameters ${\vec{m}}_{i}$ that match the measured joint torques at the ankle, knee, and hip while consuming a minimal amount of metabolic energy. This amounts to a dual objective optimization problem, with metabolic and kinetic cost functions being simultaneously minimized. The specific forms of these two costs are summarized below.

#### Metabolic cost estimation

Muscle metabolic consumption is known to depend on several factors including fascicle size, excitation level, activation level, length, velocity, force, and active force production [24]. For a given muscle in our model all of these quantities except for the excitation and activation levels depend on the muscle-tendon morphology and therefore vary across potential solutions. To model these effects we use the metabolic consumption model derived by Umberger et al [24, 34]. This function has demonstrated exceptional predictive power in a number of applications and is the most widely-accepted metabolic cost measure available. It expresses metabolic power per unit muscle mass $\dot{E}$ as the sum of four terms:

$$\begin{array}{c} \dot{E}={\dot{h}}_{A}+{\dot{h}}_{M}+{\dot{h}}_{SL}+{\dot{w}}_{CE}. \end{array}$$ (13)

Here ${\dot{h}}_{A}$ is the activation heat rate, which is associated with the transport of Ca^2+^ ions from the sarcoplasmic reticulum. ${\dot{h}}_{M}$ is the maintenance heat rate; both it and the shortening/lengthening heat rate ${\dot{h}}_{SL}$ are due to actomyosin interaction. ${\dot{w}}_{CE}$ is the mechanical work rate of the contractile element, normalized to muscle mass. For a full mathematical description of these terms see [24] and the supplementary materials of [34].

To estimate full body metabolic cost, we integrate $\dot{E}$ for each muscle of the leg, multiply by each appropriate muscle mass, and sum. Bilateral symmetry was assumed as full data were collected for only one leg of each participant. For each muscle *i* the mass *M*_*i*_ was estimated using

$$\begin{array}{c} M_{i}=\frac{\rho F_{max,i}l_{opt,i}}{\sigma }, \end{array}$$ (14)

where *ρ* = 1059.7kgm^−3^ and *σ* = 0.25MPa are the average density and specific tension of skeletal muscle, respectively. The metabolism for the rest of the body (excluding leg muscles) was estimated by multiplying the remaining mass by the measured basal rate ${\dot{E}}_{bas}$ ([W/kg]) of standing. The final metabolic cost in the model *C*_*met*_ in a time window T was then

$$\begin{array}{c} C_{met}=\sum_{i}\left(M_{i}\int_{0}^{T}{\dot{E}}_{i}\left(t\right)\;dt\right)+\left(M-\sum_{i}M_{i}\right){\dot{E}}_{bas}T, \end{array}$$ (15)

where *M* is total body mass and the sums were taken over all muscles in both legs.

#### Kinetic cost function

In addition to minimizing metabolic cost, we sought solutions that most accurately reproduced the measured joint moments. We defined the corresponding cost *C*_*kin*_ as

$$\begin{array}{c} C_{kin}=1-\frac{R_{ankle}^{2}+R_{knee}^{2}+R_{hip}^{2}}{3}, \end{array}$$ (16)

where the *R*^2^ values are the coefficients of determination between the modeled and observed joint torques.

### System Identification Procedure

As mentioned above, optimal muscle-tendon parameters were identified using a dual objective optimization. Fifty morphological parameters were optimized for each subject; four morphological parameters for each of the twelve modeled muscles and two additional parameters describing the hip flexor ligament. The chosen muscle-tendon parameter bounds are shown in Table 4. The maximum isometric force (*F*_*max*_) values for each muscle were constrained to fall in a window surrounding the scaled value from SIMM. The large width of this window helped to compensate for uncertainties in the EMG normalization (via MVC values, as discussed above). The scaling factor for *l*_*slack*_ and *l*_*opt*_ was chosen to ensure that the muscle fascicle length stayed within reasonable physiological operating ranges. The bounds for *K*_*sh*_ and λ_*ref*_ were taken from [16]. The bounds for the spring constant of the hip flexor ligament was chosen so that the ligament could provide anywhere from none to all of the required hip flexion moment near toe off. The engagement angle was chosen so that the tendon could turn on with the hip no more than 10° flexed, as its physiological role is to prevent overextension.

**Table 4:** Optimization problem parameter bounds. The maximum isometric force (*F*_*max*_) values for each muscle were constrained to fall in a fairly wide window surrounding the scaled value from SIMM. The scaling factor for *l*_*slack*_ and *l*_*opt*_ was chosen to ensure that each muscle fascicle length stayed within physical operating ranges. Here the parameter *w* governs the width of the active force-length relation of the muscle; its value varies by muscle and all values are given in S2 Text. The bounds for *K*_*sh*_ and λ_*ref*_ were taken from [16]. The bounds for the spring constant of the hip flexor ligament was chosen so that the ligament could provide anywhere from none to all of the required hip flexion moment near toe off. The engagement angle was chosen so that the tendon could engage with the hip no more than 10° flexed, as its physiological role is to prevent overextension.

Parameter	Lower Bound	Upper Bound
*F*_*max*_	0.5*F_*max*,*SIMM*_	3.0*F_*max*,*SIMM*_
*l*_*sl*_,*l*_*opt*_ mult.	Ensure *l*_*m*_ < *l*_*opt*_(1 + *w*)	Ensure *l*_*m*_ ≥ *l*_*opt*_(1−*w*)
*K*_*sh*_	2	5
λ_*ref*_	0.02	0.09
*θ*_*HFL*_	−*π*/18	|min(*θ*_*hip*_)|
*K*_*HFL*_	0	2*max(*τ*_*hip*_)/|min(*θ*_*hip*_)|

The model was constructed using MATLAB and Simulink and integrated using the ode15s (stiff/NDF) solver. Computations were parallelized and carried out using the Mathworks Cloud Center, a computer cluster operated through Amazon Web Services. This resource proved useful as our runs were configured to test many solutions and took about 10 hours to run on a single CPU. The optimization algorithm employed was MATLAB’s gamultiobj, a controlled elitist genetic algorithm that is a variant of the NSGA-II algorithm [30]. This method was chosen because of the likely presence of local minima in both objectives and tuned as shown in Table 5. Optimization tunings were chosen to ensure generations sufficiently large to force the optimizer to thoroughly search the space, eliminating the need for population seeding. Each trial solution was allowed to run for two gait cycles, with only the results of the second gait cycle being considered. This removed initial transient effects and allowed evaluation of the system over the full gait cycle.

**Table 5:** Optimization settings in MATLAB.

Optimizer Setting	Value
PopulationSize	1000
EliteCount	25
Generations	100
MutationFcn	mutationadaptfeasible
CrossoverFraction	0.8
Vectorized	On
PopInitRange	Full Space

#### Choosing an optimal solution

The result of our multi-objective optimization is a Pareto Front, a set of solutions where one cost function cannot be further reduced without compromising on another. In the ideal case this front consists of one solution where all objectives are optimized; however this rarely occurs in noisy systems. Our dual objective optimization leads to a rounded Pareto Front, from which we chose one “best” solution.

As can be seen in Fig 4, our Pareto optimal solutions are composed of three regions. The solutions in the lower left represent relatively low metabolic cost but fail to track the observed joint torque profiles. The performance of these solutions is typically accounted for by small *F*_*max*_ values; minimizing these parameters causes metabolically inexpensive but weak muscle forces. In the upper right corner are solutions with excellent kinetic agreement but high metabolic costs. These solutions essentially overfit the observed torque profiles by driving one or more muscles harder than is physically reasonable. This effort is required to maximize the kinetic fit because of deficiencies in the data, particularly in the EMG measurements. The solutions that represent the human should be somewhere between these two extremes, producing a good kinetic fit at a reasonable metabolic cost.

In order to choose a solution in the biologically plausible region, we consulted the metabolic energy budget of each subject. Fig 5 shows a polynomial fit of the fraction of full body metabolic cost consumed by each modeled muscle as a function of kinetic fit (quantified by average joint torque *R*^2^) along the Pareto Front for each participant. The fractions represent the sum over both legs and take basal expenditures into account. As can be seen from the plots, the budget is fairly consistent for low to moderate kinetic fit and MCOT, then sees a few muscles begin to dominate as the high *R*^2^ and metabolic cost range is approached. Typically one to three muscles overexert themselves to force a marginally better kinetic fit, causing the metabolic cost distribution to differ significantly from more reasonable energies. Physically this would lead to fatigue of the muscle in question, or in it being modeled as much larger than its actual size (since muscle mass is taken to be proportional to *F*_*max*_). Fig 5 shows that the vastus (VAS) muscle group provides the largest contribution to metabolic cost for each participant and ramps up its fractional contribution as kinetic fit is maximized. This is caused by the large size of the vastus and the tendency of the optimizer to throw progressively more energy into it if the knee extension moment in early stance is inadequate. Given the importance of this muscle to the overall metabolic budget and its consistent ramp up, we chose to use it for selecting an optimal solution.

To find our optimal solutions, we first fit the trends in fractional metabolic cost as a function of kinetic fit with a fifth order polynomial, as shown in Fig 5. We then considered the relative change in vastus fractional metabolic consumption Δ_*VAS*_:

$$\begin{array}{c} \Delta_{VAS}=\frac{F_{VAS}\left(R^{2}\right)-F_{VAS}\left(\text{min}\left(R^{2}\right)\right)}{F_{VAS}\left(\text{max}\left(R^{2}\right)\right)-F_{VAS}\left(\text{min}\left(R^{2}\right)\right)} \end{array}$$ (17)

Here *F*_*VAS*_ represents the fraction of overall metabolic cost attributed to the vastus, as a function of the mean coefficient of determination for the kinetic fit (*R*^2^). We found that choosing Δ_*VAS*,*opt*_ = 0.63 as a cutoff allowed us to quantitatively match the experimental metabolic costs in four out of five participants and led to an average MCOT error smaller than 0.01. This cutoff is represented by the dotted blue vertical lines in Fig 5, with the vertical black lines representing the uncertainty in the experimental measurement. Both sets of vertical lines were obtained by mapping the *R*^2^ values listed on the x-axis in Fig 5 to their corresponding metabolic costs in the solutions shown in Fig 4. The chosen optimal solution was taken to be the point with maximal kinetic fit along the Pareto Front that had Δ_*VAS*_ < Δ_*VAS*,*opt*_. In Fig 4, this point is represented as a white diamond on the solution space plot of each participant. The full parameter sets for these optimal solutions are displayed in Table 6. For reference a list of frequently used abbreviations and symbols is given in Table 7.

**Table 6:** Parameter values for the chosen optimal solution for each participant.

Parameter	Participant 1	Participant 2	Participant 3	Participant 4	Participant 5
TA *F*_*max*_ (N)	477	874	523	497	617
TA *l*_*sl*_, *l*_*opt*_ scaling	0.214	0.192	0.210	0.191	0.193
TA λ_*ref*_	0.040	0.057	0.045	0.069	0.061
TA *K*_*sh*_	2.19	2.33	2.73	2.45	2.97
SOL *F*_*max*_ (N)	3974	4632	4219	1992	3858
SOL *l*_*sl*_, *l*_*opt*_ scaling	0.282	0.244	0.266	0.255	0.253
SOL λ_*ref*_	0.061	0.080	0.064	0.048	0.051
SOL *K*_*sh*_	2.90	2.98	2.51	2.76	3.31
GAS *F*_*max*_ (N)	2075	1819	1816	1616	2278
GAS *l*_*sl*_, *l*_*opt*_ scaling	0.438	0.369	0.411	0.382	0.385
GAS λ_*ref*_	0.047	0.053	0.051	0.047	0.051
GAS *K*_*sh*_	2.49	2.93	2.86	2.93	3.81
VAS *F*_*max*_ (N)	3864	3229	8141	4472	4827
VAS *l*_*sl*_, *l*_*opt*_ scaling	0.192	0.162	0.177	0.167	0.171
VAS λ_*ref*_	0.055	0.056	0.052	0.061	0.079
VAS *K*_*sh*_	2.00	2.73	3.03	2.61	2.86
BFSH *F*_*max*_ (N)	317	289	404	358	398
BFSH *l*_*sl*_, *l*_*opt*_ scaling	0.156	0.110	0.147	0.133	0.116
BFSH λ_*ref*_	0.052	0.058	0.063	0.067	0.055
BFSH *K*_*sh*_	2.15	2.40	2.85	2.60	2.48
RF *F*_*max*_ (N)	681	888	1293	511	621
RF *l*_*sl*_, *l*_*opt*_ scaling	0.408	0.353	0.381	0.369	0.371
RF λ_*ref*_	0.067	0.060	0.059	0.071	0.054
RF *K*_*sh*_	2.93	2.22	3.05	2.76	3.03
HAM *F*_*max*_ (N)	1503	1189	1694	1177	1232
HAM *l*_*sl*_, *l*_*opt*_ scaling	0.410	0.343	0.385	0.386	0.387
HAM λ_*ref*_	0.067	0.051	0.058	0.060	0.073
HAM *K*_*sh*_	2.30	2.11	2.90	2.46	2.67
ILL *F*_*max*_ (N)	713	737	991	560	1079
ILL *l*_*sl*_, *l*_*opt*_ scaling	0.103	0.119	0.118	0.105	0.107
ILL λ_*ref*_	0.067	0.061	0.057	0.047	0.059
ILL *K*_*sh*_	2.16	2.60	2.46	2.30	3.15
GMAX *F*_*max*_ (N)	1405	1452	1074	973	1035
GMAX *l*_*sl*_, *l*_*opt*_ scaling	0.109	0.114	0.125	0.115	0.110
GMAX λ_*ref*_	0.070	0.060	0.056	0.048	0.056
GMAX *K*_*sh*_	2.63	2.86	3.02	2.33	3.35
GMED *F*_*max*_ (N)	1266	1311	1376	1011	1508
GMED *l*_*sl*_, *l*_*opt*_ scaling	0.054	0.057	0.052	0.058	0.053
GMED λ_*ref*_	0.051	0.051	0.056	0.049	0.056
GMED *K*_*sh*_	2.79	2.67	3.05	2.45	3.14
ADDL *F*_*max*_ (N)	403	353	447	337	628
ADDL *l*_*sl*_, *l*_*opt*_ scaling	0.119	0.115	0.105	0.097	0.103
ADDL λ_*ref*_	0.048	0.060	0.057	0.042	0.050
ADDL *K*_*sh*_	2.19	2.00	2.99	2.67	3.80
ADDM *F*_*max*_ (N)	805	874	851	758	1054
ADDM *l*_*sl*_, *l*_*opt*_ scaling	0.138	0.109	0.131	0.126	0.127
ADDM λ_*ref*_	0.069	0.053	0.055	0.044	0.053
ADDM *K*_*sh*_	2.28	2.59	2.80	2.43	3.61
*K*_*HFL*_ (Nm)	193	142	567	128	319
*θ*_0,*HFL*_ (rad)	0.040	-0.023	-0.050	0.045	0.006

**Table 7:** Frequently Used Abbreviations and Symbols.

Name	Meaning
*F*_*max*_	Maximal muscle isometric force
*l*_*sl*_	Tendon slack length
*l*_*opt*_	Optimal muscle length
*K*_*sh*_	Tendon force-length shape factor
λ_*ref*_	Tendon reference strain
*K*_*HFL*_	Iliofemoral/hip flexor ligament spring constant
*θ*_0,*HFL*_	Hip flexor ligament engagement angle
*l*_*m*_, *l*_*ce*_	Muscle fascicle/contractile element length (same)
*v*_*m*_, *v*_*ce*_	Muscle fascicle/contracitle element velocity (same)
*v*_*max*_	Maximum muscle fascicle velocity
*θ*(*t*)	Muscle pennation angle as a function of time
*l*_*mHS*_	Muscle fascicle length at heel strike
$\vec{m}$	Morphological parameter vector
$\dot{E}$	Metabolic consumption
*x*(*t*)	Muscle excitation
*a*(*t*)	Muscle activation
*r*(*t*)	Muscle moment arm
*τ*(*t*)	Torque
*R*^2^	Coefficient of determination
TA	Tibialis anterior
SOL	Soleus
GAS	Gastrocnemius
VAS	Vastus
BFSH	Biceps femoris short head
RF	Rectus femoris
HAM	Hamstring
ILL	Illiacus
ADDL	Adductor Longus
ADDM	Adductor Magnus
HAB	Hip abductors
GLU	Gluteus maximus
GC	Gait cycle
EMG	Electromyography
MVC	Maximal Voluntary Contraction
MCOT	Metabolic Cost of Transport
MTU/MTC	Muscle tendon unit/complex (same)

## Supporting Information

S1 Text Addressing missing EMG data. Discusses the methods employed when EMG data (particularly in the muscles spanning the hip) were not adequate for analysis.
(PDF)

S2 Text Hill-type muscle dynamics. Provides the mathematical details of the modeled contraction dynamics as well as muscle-specific parameters.
(PDF)

S3 Text Choosing an optimal solution. Describes the method used to pick one optimal solution for each dual objective optimization problem and provides the resulting optimal muscle-tendon parameters for each participant.
(PDF)
