# Supplementary material for: Human Leg Model Predicts Muscle Forces, States, and Energetics during Walking
Source: PLoS Comput Biol. 2016 May 13;12(5):e1004912. doi: 10.1371/journal.pcbi.1004912 (PMC4866735; doi:10.1371/journal.pcbi.1004912)
Supplement: S1 Text — Discusses the methods employed when EMG data (particularly in the muscles spanning the hip) were not adequate for analysis. (PDF) [file pcbi.1004912.s001.pdf]

Supplementary Text 1: Addressing Missing EMG Data

As mentioned in the main text, full EMG data sets were difficult to obtain. This was largely due to the inherent difficulty of collecting surface EMG signals in the muscles spanning the hip. In this area the muscle geometry is more complex and the muscles are buried deeper beneath the skin than in more distal regions. The hip area is also less easy to access and more prone to motion artifacts, increasing the probability of poor measurements.

In tables 1 and 2 we show, respectively, the characteristics of all participants for whom EMG signals were recorded and the muscles for which reasonable profiles were obtained. Subjects 1-5 are those described in the main text, with subjects 6-9 being other participants with EMG collected using the same protocol but excluding the ADDL, ADDM, and GMED muscle groups. Using all muscles with reasonable EMG trajectories across subjects, we formed average neural excitation and activation profiles for each muscle, as shown in Figures 1 and 2. When performing the system identification optimization described in the main text for a given subject, each unreasonable activation profile was replaced by the average activation profile for that muscle. As can be seen from Table 2 this was a relatively rare occurrence for the muscles spanning the ankle and knee but was frequently required at the hip. This lack of quality data led to our decision to substitute the excitation profiles from [1] for the muscles of the hip.

To justify replacement with average profiles we performed a Leave One Out Cross Validation (LOOCV) analysis on the reasonable excitation and activation trajectories for each muscle. We computed the Pearson r coefficient between each individual profile and the average of the remaining reasonable profiles. The average value of these r coefficients for each muscle is shown in Table 3. The values are, as expected, better for the more distal muscles and those where there is only one typical behavior. The muscles spanning the hip do not perform as well, except for GMED. Hence the excitation values for the muscles spanning the hip were replaced with the profiles in [1] (black lines in Figure 1) while other muscles used the collected data with average trajectories being substituted in the rare event they were needed. Some r values for included average profiles were small, but this likely did not impact the analysis significantly because these activation signals were typically small in magnitude.

References

[1] Jacquelin Perry. *Gait Analysis*. SLACK Incorporated, Thorofare, NJ 08086-9447, first edition, 1992.

Subject	Age	Mass	Leg Length	Ethnicity	Fav. Sport	Min. MCOT
1	25	65.4 kg	1.028 m	Caucasian	Running	1.47 m/s
2	26	65.0 kg	0.902 m	Caucasian	Running	1.38 m/s
3	24	80.3 kg	0.953 m	African	Basketball	1.32 m/s
4	29	68.9 kg	0.933 m	Caucasian	Running	1.31 m/s
5	24	72.3 kg	0.927 m	Caucasian	Running	1.49 m/s
6	24	63.9 kg	0.927 m	Caucasian	Running	1.40 m/s
7	22	66.3 kg	0.923 m	Polish/Chinese	Bicycling	1.50 m/s
8	27	78.8 kg	0.944 m	Caucasian	Football	1.20 m/s
9	24	80.2 kg	0.980 m	African	Soccer	1.00 m/s

Table 1: Relevant characteristics of all participants from whom EMG data were recorded. Note that Min. MCOT Speed was only estimated from metabolic data for subjects 1-5. It was estimated using subject preference for participants 6-9.

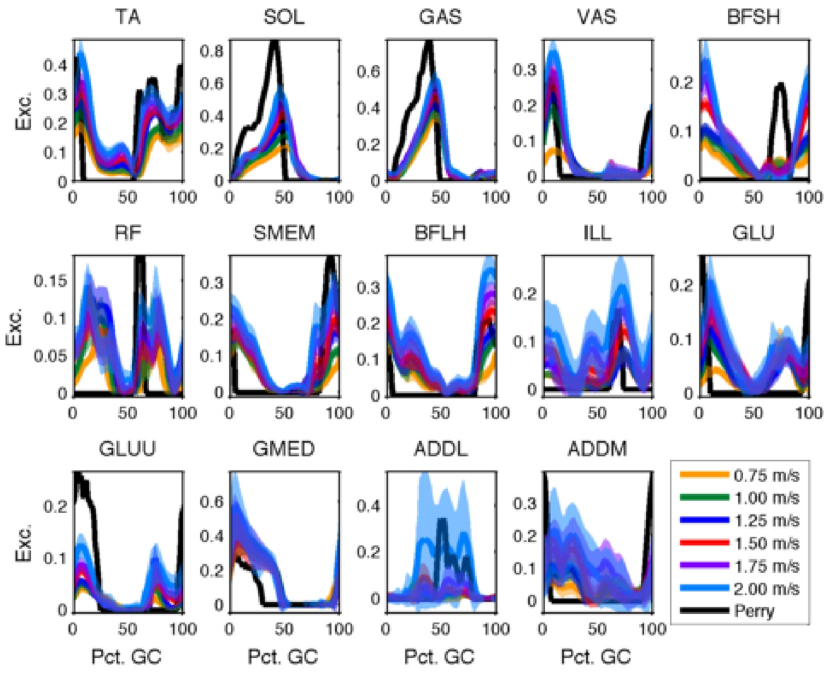

Figure 1: Mean neural excitation trajectories across speed for all subjects with reasonable profiles. The estimates were obtained via the Sanger Bayesian method and are compared to the literature values in Perry's *Gait Analysis* [1].

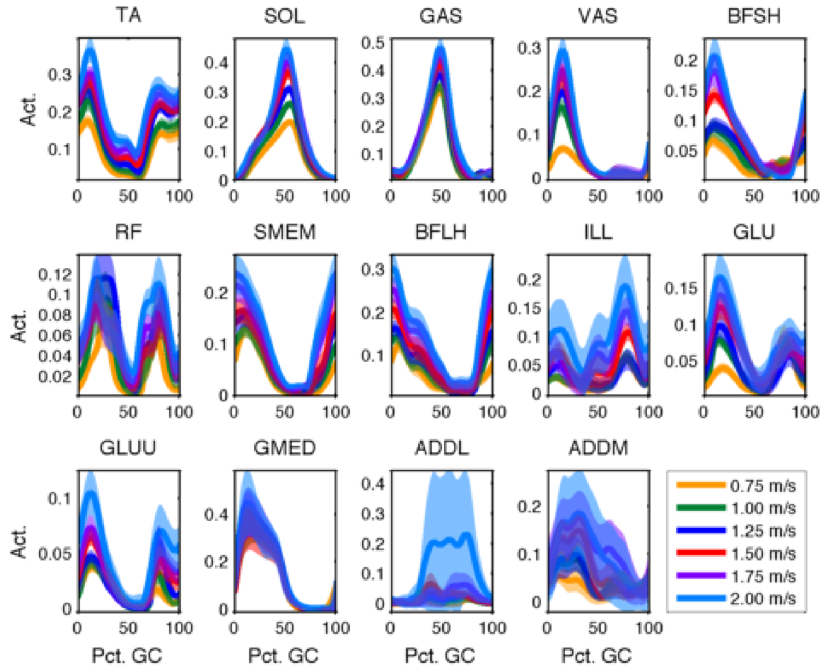

Figure 2: Mean activation trajectories across speed for all subjects with reasonable profiles. The estimates were obtained using the Sanger Bayesian method coupled with a filter representing activation dynamics.

Subject	Reasonable EMG Profiles
1	TA, SOL, GAS, VAS, BFSH, RF, HAM, GMAX, GMED
2	TA, GAS, VAS, BFSH, RF, HAM, GMED, ADDM
3	SOL, GAS, VAS, BFSH, RF, HAM, ILL, GMAX, GMED, ADDL, ADDM
4	TA, SOL, GAS, VAS, BFSH, HAM, ILL, GMED
5	TA, SOL, GAS, VAS, HAM, ILL, GMED
6	TA, SOL, GAS, VAS, BFSH, RF, HAM, ILL, GMAX
7	TA, SOL, GAS, VAS, BFSH, RF, HAM, ILL, GMAX
8	TA, SOL, GAS, VAS, BFSH, RF, HAM, GMAX, GMED
9	TA, SOL, GAS, VAS, BFSH, RF, HAM, GMAX

Table 2: Muscles with reasonable EMG profiles for each subject. Note that GMED, ADDL, and ADDM were not collected on subjects 6, 7, 8, or 9.

Muscle	Mean Excitation LOOCV r Value	Mean Activation LOOCV r Value
TA	[0.74 0.76 0.82 0.80 0.78 0.76]	[0.76 0.79 0.85 0.82 0.80 0.77]
SOL	[0.96 0.97 0.97 0.96 0.95 0.91]	[0.97 0.98 0.98 0.98 0.97 0.94]
GAS	[0.93 0.93 0.92 0.93 0.92 0.92]	[0.95 0.95 0.95 0.95 0.95 0.94]
VAS	[0.79 0.91 0.92 0.91 0.92 0.94]	[0.80 0.93 0.94 0.93 0.94 0.95]
BFSH	[0.52 0.58 0.59 0.72 0.75 0.81]	[0.55 0.64 0.64 0.77 0.81 0.86]
RF	[0.49 0.50 0.44 0.36 0.39 0.42]	[0.52 0.55 0.47 0.37 0.37 0.39]
HAM	[0.83 0.85 0.79 0.76 0.76 0.76]	[0.86 0.89 0.84 0.80 0.82 0.83]
ILL	[0.46 0.57 0.14 0.58 0.41 0.27]	[0.51 0.65 0.14 0.66 0.54 0.33]
GMAX	[0.45 0.57 0.74 0.70 0.73 0.60]	[0.45 0.61 0.79 0.6 0.79 0.65]
GMED	[0.81 0.73 0.59 0.62 0.61 0.62]	[0.84 0.75 0.60 0.67 0.66 0.64]
ADDL	N/A	N/A
ADDM	[-0.24 0.05 0.37 0.33 0.39 0.29]	[-0.31 0.01 0.36 0.37 0.37 0.33]

Table 3: r values for LOOCV. The six values in each array refer to walking at 0.75 m/s, 1.00 m/s, 1.25 m/s, 1.50 m/s, 1.75 m/s, and 2.00 m/s, respectively. No values were computed for ADDL because there was only one set of reasonable measurements of that muscle.
