# Supplementary material for: Human Leg Model Predicts Muscle Forces, States, and Energetics during Walking
Source: PLoS Comput Biol. 2016 May 13;12(5):e1004912. doi: 10.1371/journal.pcbi.1004912 (PMC4866735; doi:10.1371/journal.pcbi.1004912)
Supplement: S2 Text — Provides the mathematical details of the modeled contraction dynamics as well as muscle-specific parameters. (PDF) [file pcbi.1004912.s002.pdf]

Supplementary Text 2: Hill-Type Muscle Dynamics

In this study, muscle contraction dynamics are modeled using a Hill-type formulation similar to that of Geyer and Herr[1]. The muscle model contains a contractile element (CE) that represents the active muscle fibers and a parallel elasticity (PE) that represents the elastic structures surrounding the muscle. The force produced by the contractile element is

$$F_{CE} = \alpha F_{max} f_l(l_{CE}) f_v(v_{CE}), \tag{1}$$

where α is the muscle activation, l_{CE} is the contractile element length, $v_{CE} = \dot{l}_{CE}$ is the contractile element velocity, and F_{max} is the maximum isometric force. $f_l(l_{CE})$ and $f_v(v_{CE})$ are the active force-length and force-velocity relations and are given by

$$f_l(l_{CE}) = \frac{-1}{w^2}\left(\frac{l_{CE}}{l_{opt}}\right)^2 + \frac{2}{w^2}\left(\frac{l_{CE}}{l_{opt}}\right) - \frac{1}{w^2} + 1 \tag{2}$$

and

$$f_v(v_{CE}) = \begin{cases} \frac{v_{max}+v_{CE}}{v_{max}-Kv_{CE}}, & v_{CE} < 0 \\ N - \frac{(N-1)(v_{max}-v_{CE})}{7.56Kv_{CE}+v_{max}}, & v_{CE} \geq 0. \end{cases} \tag{3}$$

In (2) l_{opt} is the fascicle length where maximal active force is produced and w determines the width of the active force-length relation. In (3) v_{max} is the maximal muscle velocity, K is a curvature constant, and $N = 1.5$ is the muscle force (in units of F_{max}) at the muscle's maximum lengthening velocity. The active force-length relation (2) comes from [2] and was chosen because, while similar to the relation in [1], it allows for scaling based on fiber composition. The parallel elasticity produces force according to

$$F_{PE}(l_{CE}) = \begin{cases} F_{max}\left(\frac{l_{CE}-l_{opt}}{l_{opt}w}\right)^2, & l_{CE} \geq l_{opt} \\ 0, & l_{CE} < l_{opt}. \end{cases} \tag{4}$$

Note that this quadratic elastic element is only engaged for $l_{CE} \geq l_{opt}$. Finally, as in [1], we include a buffer elasticity

$$F_{BE}(l_{CE}) = \begin{cases} F_{max}\frac{2}{w}\left(\frac{l_{CE}-l_{opt}(1-w)}{l_{opt}}\right)^2, & l_{CE} \leq l_{opt}(1-w) \\ 0 & l_{CE} > l_{opt}(1-w) \end{cases} \tag{5}$$

that prevents the muscle fascicle from shortening excessively. It is a rarely engaged numerical tool and does not reflect a physical characteristic of muscle. The total force produced by the muscle fascicle is then

$$F(\alpha, l_{CE}, v_{CE}) = F_{CE}(\alpha, l_{CE}, v_{CE}) + F_{PE}(l_{CE}) - F_{BE}(l_{CE}). \tag{6}$$

The inputs α, l_{CE}, and v_{CE} all vary with time, and $l_{CE}(t)$ represents the state variable of the model. The differential equations were solved in a manner based off of [1] (source code available at link in reference).

Muscle-specific parameters used in these muscle force computations are given in Table 1. Muscle fiber compositions, w, and $\{\tau_{act}, \tau_{deact}\}$ (the activation and deactivation time constants for the muscle excitation to activation filter) were taken from [3] and its supplementary material. The curvature constant K used in the force-velocity relation is computed via

$$K = 8(1 - \text{FFT}), \tag{7}$$

Muscle	Percent FT	τ_{act} [ms]	τ_{deact} [ms]	w	K	v_{max} [l_{opt}/s]	θ_0
TA	25%	68	76	0.49	6.60	6.0	5°
SOL	20%	71	79	0.80	6.24	6.4	25°
GAS	50%	57	62	0.61	8.40	4.0	17°
VAS	50%	57	62	0.55	8.40	4.0	5°
BFSH	35%	64	70	0.75	8.40	5.2	23°
RF	65%	49	65	0.76	9.48	2.8	5°
HAM	35%	64	70	0.75	7.32	5.2	15°
ILL	50%	57	62	0.74	8.40	4.0	7°
GMAX	45%	59	65	0.77	8.04	4.4	0°
GMED	50%	57	62	0.77	8.40	4.0	19°
ADDL	35%	64	70	0.74	7.32	5.2	6°
ADDM	45%	59	65	0.75	8.04	4.4	3°

Table 1: Muscle-specific model parameters. Muscle fiber compositions, w, τ_{act}, and τ_{deact} were taken from [3] and its supplementary material.

where FFT is the fraction of fast twitch fiber in the muscle. The maximal velocity v_{max} is computed using

$$v_{max} = 4.8(1 + 1.5\text{FFT}). \tag{8}$$

These two equations come from mapping the formulae for the normalized Hill constants A_{REL} and B_{REL} in terms of FFT (Equations (1) and (2) of [4]) to K and v_{max}.

References

1. Geyer H, Herr H. A muscle-reflex model that encodes principles of legged mechanics produces human walking dynamics and muscle activities. IEEE transactions on neural systems and rehabilitation engineering : a publication of the IEEE Engineering in Medicine and Biology Society. 2010 Jun;18(3):263–73. Available from: http://www.ncbi.nlm.nih.gov/pubmed/20378480. Code available from: https://www.cs.cmu.edu/ hgeyer/Software/Neuromuscular%20Model/Geyer%20Neuromuscular%20Model.zip.

2. van Soest AJ, Bobbert MF. The contribution of muscle properties in the control of explosive movements. Biological Cybernetics 1993 (69): 195–204.

3. Umberger BR. Stance and swing phase costs in human walking. Journal of the Royal Society, Interface / the Royal Society. 2010 Sep;7(50):1329–40. Available from: http://www.pubmedcentral.nih.gov/articlerender.fcgi?artid=2894890&tool=pmcentrez&rendertype=abstract.

4. Umberger BR, Gerritsen KGM, Martin PE. A model of human muscle energy expenditure. Computer methods in biomechanics and biomedical engineering. 2003 Apr;6(2):99–111. Available from: http://www.ncbi.nlm.nih.gov/pubmed/12745424.
