# Supplementary material for: Human Leg Model Predicts Muscle Forces, States, and Energetics during Walking
Source: PLoS Comput Biol. 2016 May 13;12(5):e1004912. doi: 10.1371/journal.pcbi.1004912 (PMC4866735; doi:10.1371/journal.pcbi.1004912)
Supplement: S3 Text — Describes the method used to pick one optimal solution for each dual objective optimization problem and provides the resulting optimal muscle-tendon parameters for each participant. (PDF) [file pcbi.1004912.s003.pdf]

# Supplementary Text 3: Choosing an Optimal Solution

In multi-objective optimization problems, the Pareto Front is the set of solutions where one cost function cannot be further reduced without compromising on another. In the ideal case this front consists of one solution where all objectives are optimized; however this rarely occurs in noisy systems. Our dual objective optimization procedure leads to a rounded Pareto Front, from which we need to choose one "best" solution.

As can be seen in Figure 1, our Pareto optimal solutions are composed of three regions. The solutions in the lower left represent relatively low metabolic cost but fail to track the observed joint torque profiles. The performance of these solutions is typically accounted for by small $F_{max}$ values; minimizing these parameters causes metabolically inexpensive but weak muscle forces. In the upper right corner are solutions with excellent kinetic agreement but high metabolic costs. These solutions essentially overfit the observed torque profiles by driving one or more muscles harder than is physically reasonable. This effort is required to maximize the kinetic fit because of deficiencies in the data, particularly in the EMG measurements. The solutions that represent the human should be somewhere between these two extremes, producing a good kinetic fit at a reasonable metabolic cost.

In order to choose a solution in the biologically plausible region, we consulted the metabolic energy budget of each subject. Figure 2 shows the fraction of full body metabolic cost consumed by each modeled muscle as a function of kinetic fit (quantified by average joint torque $R^2$) along the Pareto Front for each participant. The fractions represent the sum over both legs and take basal expenditures into account. As can be seen from the plots, the budget is fairly consistent for low to moderate kinetic fit and MCOT, then sees a few muscles begin to dominate as the high $R^2$ and metabolic cost range is approached. Typically one to three muscles overexert themselves to force a marginally better kinetic fit, causing the metabolic cost distribution to differ significantly from more reasonable energies. Physically this would lead to fatigue of the muscle in question, or in it being modeled as much larger than its actual size (since muscle mass is taken to be proportional to $F_{max}$). Figure 2 also shows that the vastus (VAS) muscle group provides the largest contribution to metabolic cost for each participant and ramps up its fractional contribution as kinetic fit is maximized. This is caused by the large size of the vastus and the tendency of the optimizer to throw progressively more energy into it if the knee extension moment in early stance is inadequate. Given the importance of this muscle to the overall metabolic budget and its consistent ramp up, we chose to use it for selecting an optimal solution.

To find our optimal solutions, we first fit the trends in fractional metabolic cost as a function of kinetic fit with a fifth order polynomial, as shown in Figure 3. We then considered the relative change in vastus fractional metabolic consumption $\Delta_{VAS}$:

$$\Delta_{VAS} = \frac{F_{VAS}(R^2) - F_{VAS}(\min(R^2))}{F_{VAS}(\max(R^2)) - F_{VAS}(\min(R^2))} \tag{1}$$

Here $F_{VAS}$ represents the fraction of overall metabolic cost attributed to the vastus, as a function of the mean coefficient of determination for the kinetic fit ($R^2$). We found that choosing $\Delta_{VAS,opt} = 0.63$ as a cutoff allowed us to quantitatively match the empirical metabolic costs in four out of five participants and led to an average MCOT error smaller than 0.01. This cutoff is represented by the dotted blue vertical lines in Figure 3, with the vertical black lines representing the uncertainty on the empirical measurement. The chosen optimal solution was taken to be the point with maximal kinetic fit along the Pareto Front that had $\Delta_{VAS} < \Delta_{VAS,opt}$. In Figure 4, this point is represented as a white diamond on the solution space plot of each participant. The full parameter sets for these optimal solutions are displayed in Table 1.

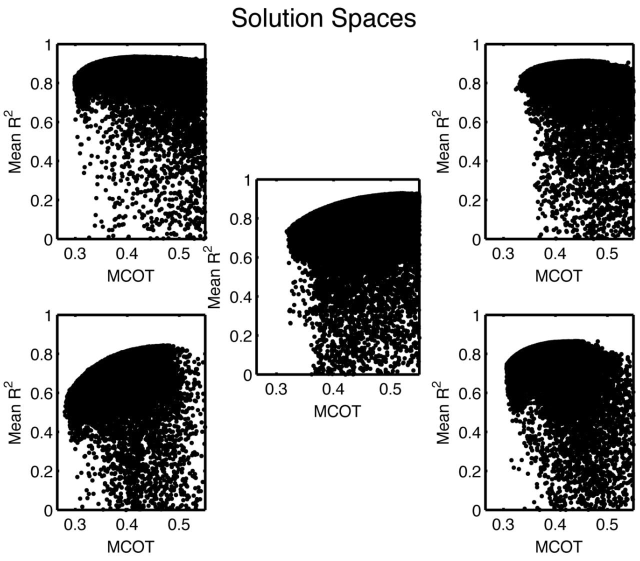


Figure 1: Solution space for each participant.

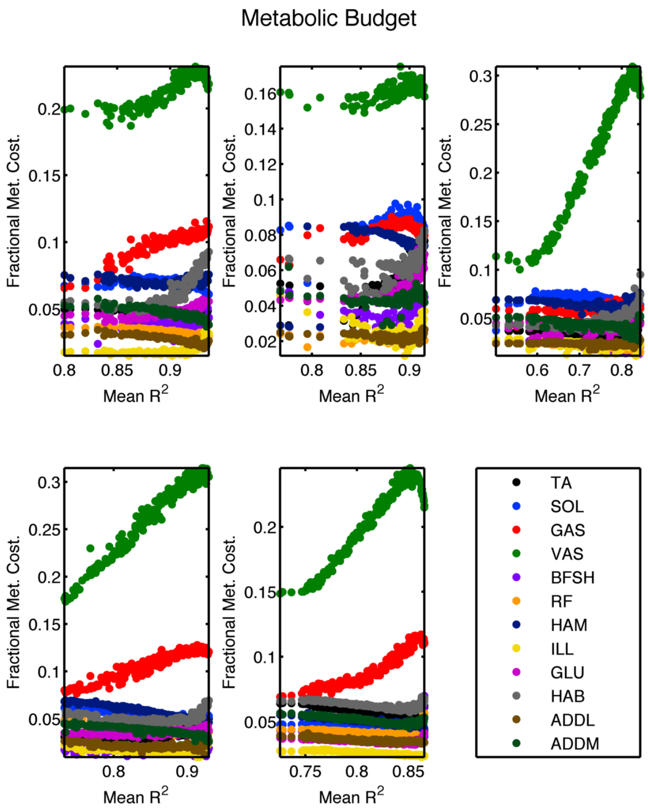


Figure 2: Per-muscle fractional metabolic cost as a function of kinetic fit for all points along the Pareto Front. Each plot represents one study participant.

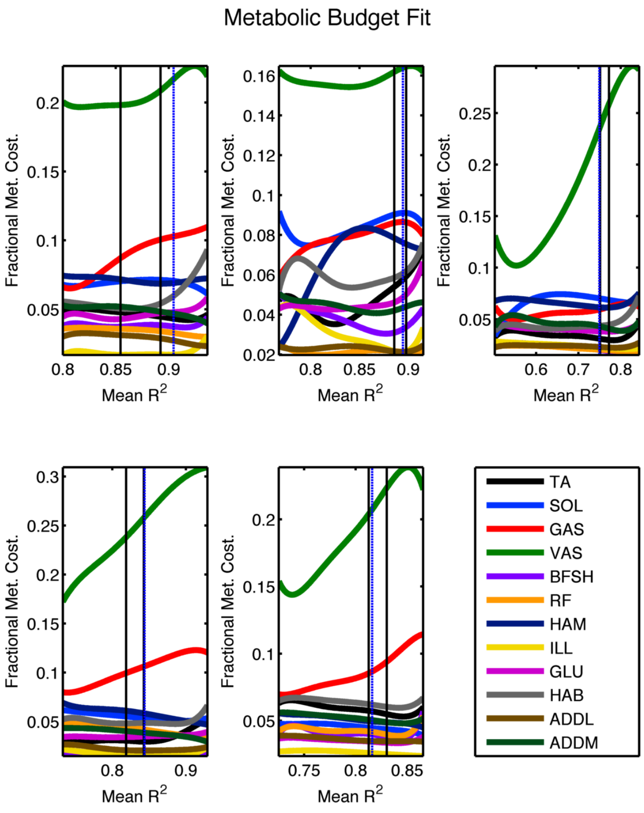


Figure 3: Polynomial fit to the per-muscle fractional metabolic cost as a function of kinetic fit along the Pareto Front, for each participant. The black lines are bounds on the observed metabolic cost while the dotted blue lines represent the locations of the chosen optimal solutions.

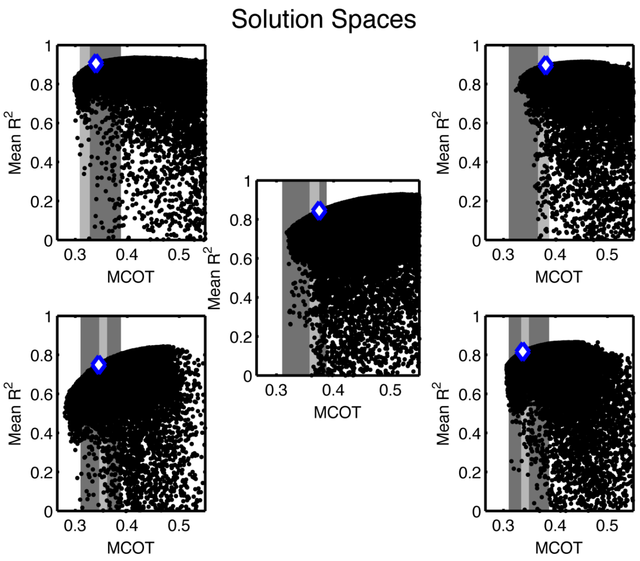


Figure 4: Best solution for each participant and its relation to measured MCOT (light gray band) and MCOT range for all participants (dark gray band).

| Parameter | Participant 1 | Participant 2 | Participant 3 | Participant 4 | Participant 5 |
|---|---|---|---|---|---|
| TA $F_{max}$ (N) | 477 | 874 | 523 | 497 | 617 |
| TA $l_{sl}$, $l_{opt}$ scaling | 0.214 | 0.192 | 0.210 | 0.191 | 0.193 |
| TA $\lambda_{ref}$ | 0.040 | 0.057 | 0.045 | 0.069 | 0.061 |
| TA $K_{sh}$ | 2.19 | 2.33 | 2.73 | 2.45 | 2.97 |
| SOL $F_{max}$ (N) | 3974 | 4632 | 4219 | 1992 | 3858 |
| SOL $l_{sl}$, $l_{opt}$ scaling | 0.282 | 0.244 | 0.266 | 0.255 | 0.253 |
| SOL $\lambda_{ref}$ | 0.061 | 0.080 | 0.064 | 0.048 | 0.051 |
| SOL $K_{sh}$ | 2.90 | 2.98 | 2.51 | 2.76 | 3.31 |
| GAS $F_{max}$ (N) | 2075 | 1819 | 1816 | 1616 | 2278 |
| GAS $l_{sl}$, $l_{opt}$ scaling | 0.438 | 0.369 | 0.411 | 0.382 | 0.385 |
| GAS $\lambda_{ref}$ | 0.047 | 0.053 | 0.051 | 0.047 | 0.051 |
| GAS $K_{sh}$ | 2.49 | 2.93 | 2.86 | 2.93 | 3.81 |
| VAS $F_{max}$ (N) | 3864 | 3229 | 8141 | 4472 | 4827 |
| VAS $l_{sl}$, $l_{opt}$ scaling | 0.192 | 0.162 | 0.177 | 0.167 | 0.171 |
| VAS $\lambda_{ref}$ | 0.055 | 0.056 | 0.052 | 0.061 | 0.079 |
| VAS $K_{sh}$ | 2.00 | 2.73 | 3.03 | 2.61 | 2.86 |
| BFSH $F_{max}$ (N) | 317 | 289 | 404 | 358 | 398 |
| BFSH $l_{sl}$, $l_{opt}$ scaling | 0.156 | 0.110 | 0.147 | 0.133 | 0.116 |
| BFSH $\lambda_{ref}$ | 0.052 | 0.058 | 0.063 | 0.067 | 0.055 |
| BFSH $K_{sh}$ | 2.15 | 2.40 | 2.85 | 2.60 | 2.48 |
| RF $F_{max}$ (N) | 681 | 888 | 1293 | 511 | 621 |
| RF $l_{sl}$, $l_{opt}$ scaling | 0.408 | 0.353 | 0.381 | 0.369 | 0.371 |
| RF $\lambda_{ref}$ | 0.067 | 0.060 | 0.059 | 0.071 | 0.054 |
| RF $K_{sh}$ | 2.93 | 2.22 | 3.05 | 2.76 | 3.03 |
| HAM $F_{max}$ (N) | 1503 | 1189 | 1694 | 1177 | 1232 |
| HAM $l_{sl}$, $l_{opt}$ scaling | 0.410 | 0.343 | 0.385 | 0.386 | 0.387 |
| HAM $\lambda_{ref}$ | 0.067 | 0.051 | 0.058 | 0.060 | 0.073 |
| HAM $K_{sh}$ | 2.30 | 2.11 | 2.90 | 2.46 | 2.67 |
| ILL $F_{max}$ (N) | 713 | 737 | 991 | 560 | 1079 |
| ILL $l_{sl}$, $l_{opt}$ scaling | 0.103 | 0.119 | 0.118 | 0.105 | 0.107 |
| ILL $\lambda_{ref}$ | 0.067 | 0.061 | 0.057 | 0.047 | 0.059 |
| ILL $K_{sh}$ | 2.16 | 2.60 | 2.46 | 2.30 | 3.15 |
| GMAX $F_{max}$ (N) | 1405 | 1452 | 1074 | 973 | 1035 |
| GMAX $l_{sl}$, $l_{opt}$ scaling | 0.109 | 0.114 | 0.125 | 0.115 | 0.110 |
| GMAX $\lambda_{ref}$ | 0.070 | 0.060 | 0.056 | 0.048 | 0.056 |
| GMAX $K_{sh}$ | 2.63 | 2.86 | 3.02 | 2.33 | 3.35 |
| GMED $F_{max}$ (N) | 1266 | 1311 | 1376 | 1011 | 1508 |
| GMED $l_{sl}$, $l_{opt}$ scaling | 0.054 | 0.057 | 0.052 | 0.058 | 0.053 |
| GMED $\lambda_{ref}$ | 0.051 | 0.051 | 0.056 | 0.049 | 0.056 |
| GMED $K_{sh}$ | 2.79 | 2.67 | 3.05 | 2.45 | 3.14 |
| ADDL $F_{max}$ (N) | 403 | 353 | 447 | 337 | 628 |
| ADDL $l_{sl}$, $l_{opt}$ scaling | 0.119 | 0.115 | 0.105 | 0.097 | 0.103 |
| ADDL $\lambda_{ref}$ | 0.048 | 0.060 | 0.057 | 0.042 | 0.050 |
| ADDL $K_{sh}$ | 2.19 | 2.00 | 2.99 | 2.67 | 3.80 |
| ADDM $F_{max}$ (N) | 805 | 874 | 851 | 758 | 1054 |
| ADDM $l_{sl}$, $l_{opt}$ scaling | 0.138 | 0.109 | 0.131 | 0.126 | 0.127 |
| ADDM $\lambda_{ref}$ | 0.069 | 0.053 | 0.055 | 0.044 | 0.053 |
| ADDM $K_{sh}$ | 2.28 | 2.59 | 2.80 | 2.43 | 3.61 |
| $K_{HFL}$ (Nm) | 193 | 142 | 567 | 128 | 319 |
| $\theta_{0,HFL}$ (rad) | 0.040 | -0.023 | -0.050 | 0.045 | 0.006 |

Table 1: Parameter values for the chosen optimal solution for each participant.
